# Supplementary material for: Quinoa Phenotyping Methodologies: An International Consensus
Source: Plants (Basel). 2021 Aug 24;10(9):1759. doi: 10.3390/plants10091759 (PMC8472428; doi:10.3390/plants10091759)
Supplement: Supplementary file 1 [file plants-10-01759-s001.zip › Supplementary file 1_Phenotyping cards.pdf]

Supplementary file 1: Phenotyping cards

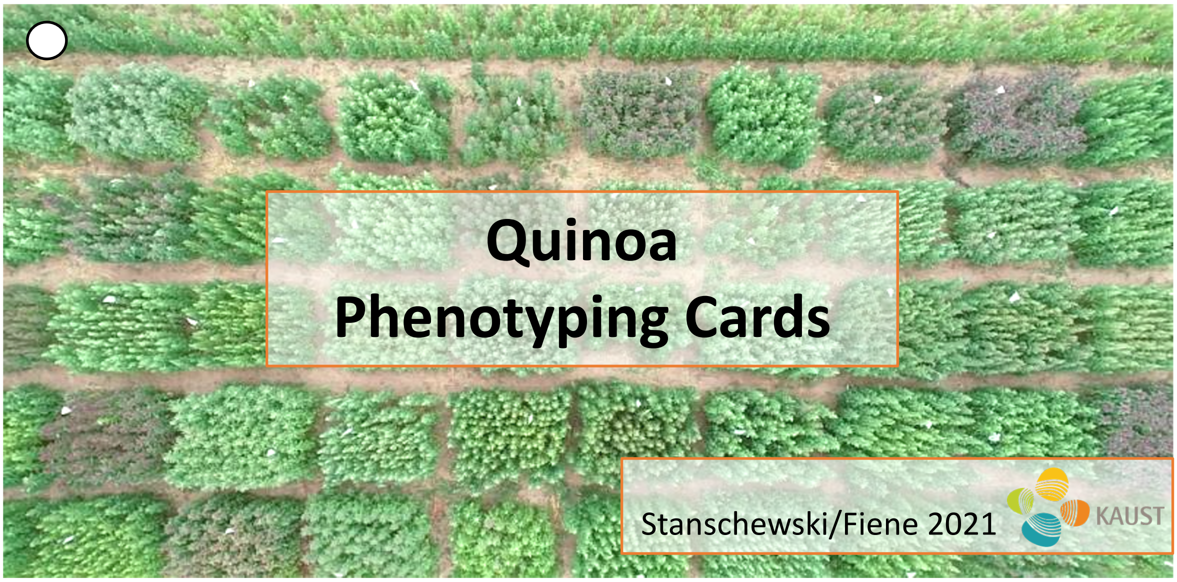

Plot population homogeneity

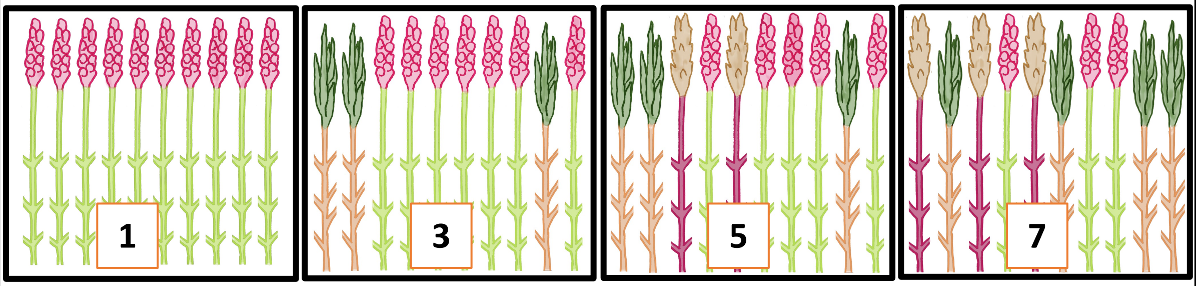

Most plants are the same (up to 10% different)	10 to 30% of plants different	30 to 50% of plants different (less than half are different)	Over 50% of plants are different (completely mixed)

Growth habit

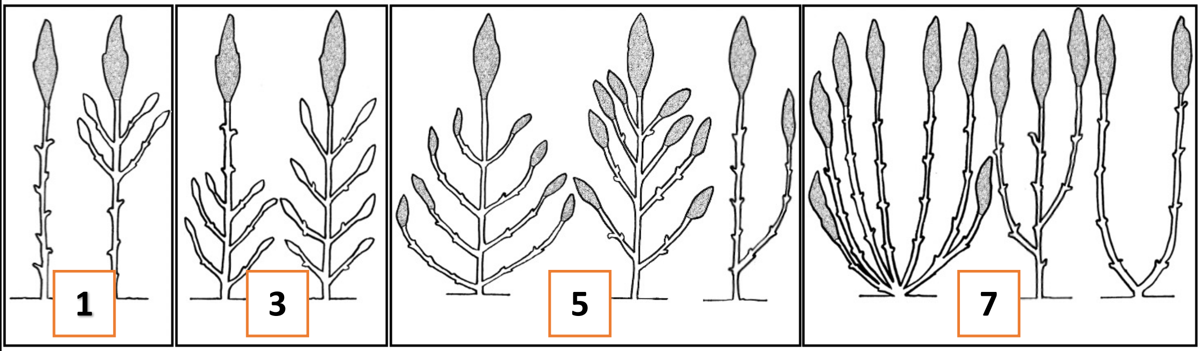

Not branched at base	Some branching from base, no significant panicles	Branching from base with more significant panicles	Main panicle can’t be identified

Growth habit examples

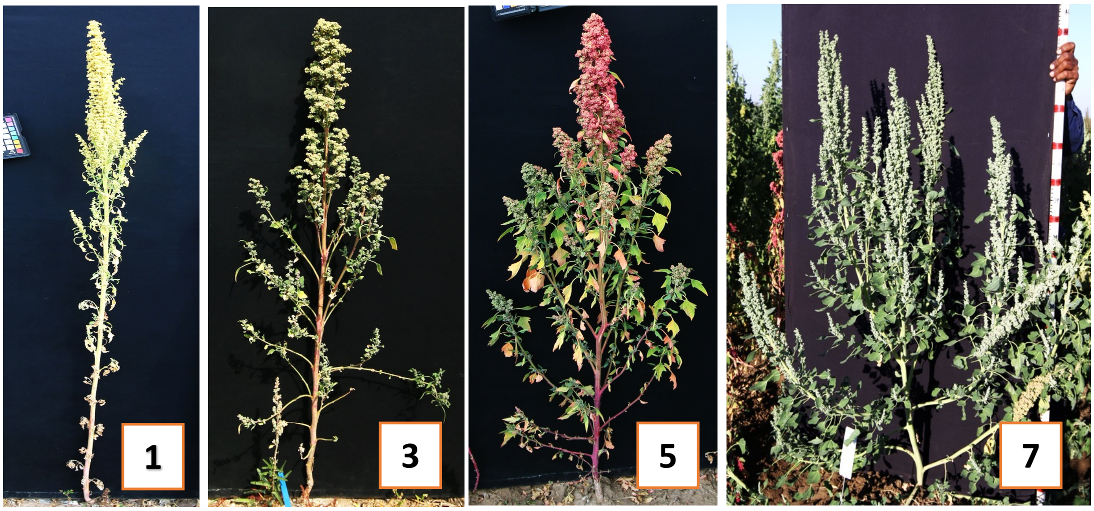

Stem breakage

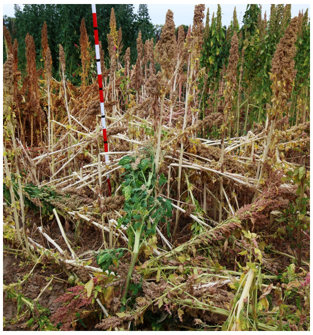

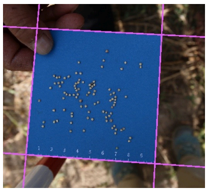

Seeds

Stem lodging

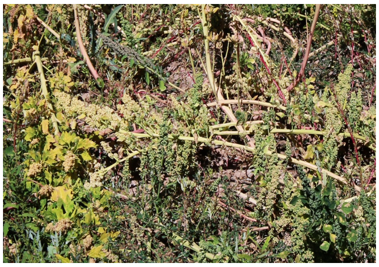

Stem lying

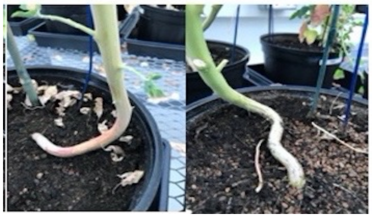

Percentage of the plot affected

1- up to 20%

3 ~ 30% (20% – 40%)

5 ~ 50% (40% – 60%)

7 ~ 70% (60% – 80%)

9 - over 80 %

Stem angle

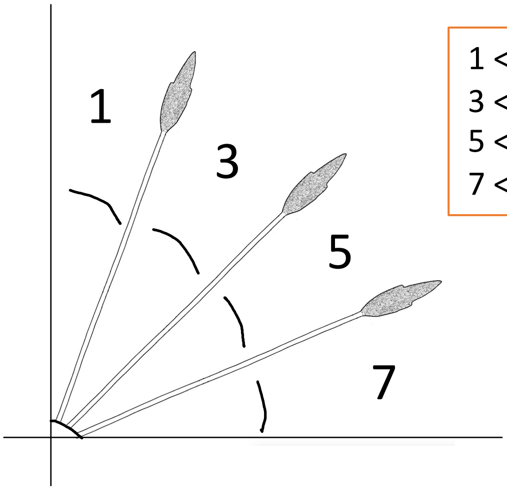

1 < 22.5°
3 < 45°
5 < 67.5°
7 < 90°

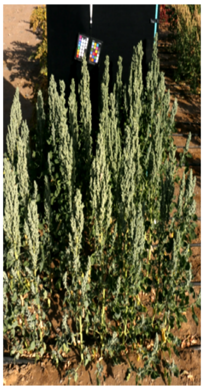

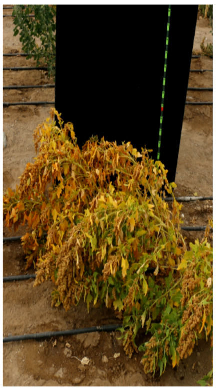

Erectness of the stems of the majority of the plot:

1 = "soldiers" to

7 = plants are severely lodged and on or near the ground

Panicle axis angle

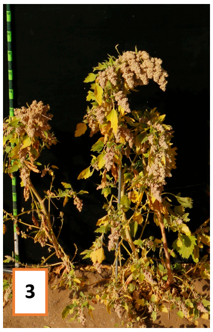

1 < 45°
3 < 90°
5 < 135°
7 < 180°

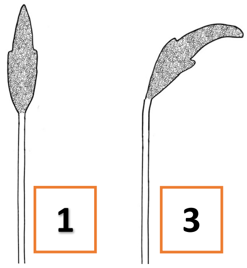

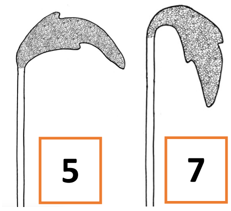

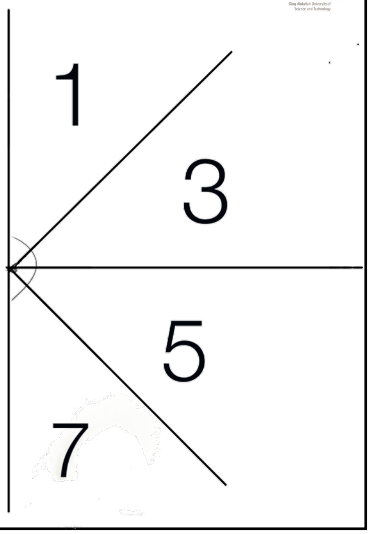

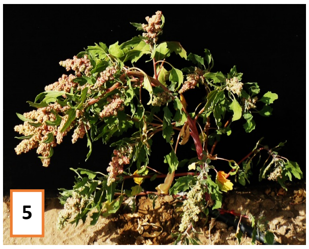

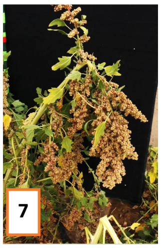

Panicle shape

1

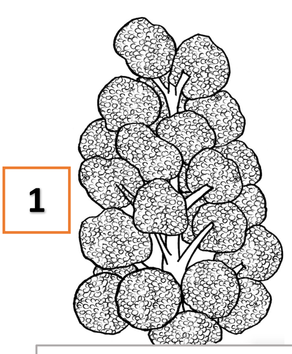

Glomerulate: glomerules with globose shape– "**bulbous clusters**"

3

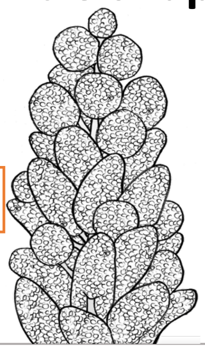

Intermediate: panicles express both amarantiform and glomerulate traits

5

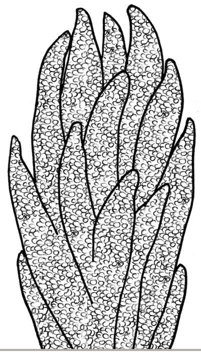

Amarantiform: glomerules with elongated shape – "**fingers**"

Panicle shape

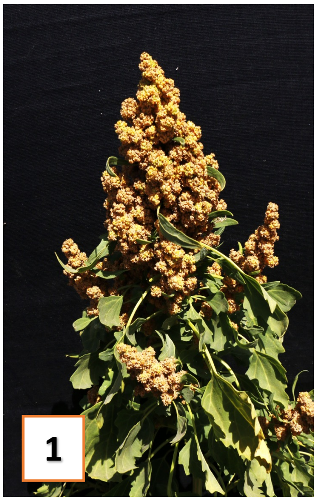

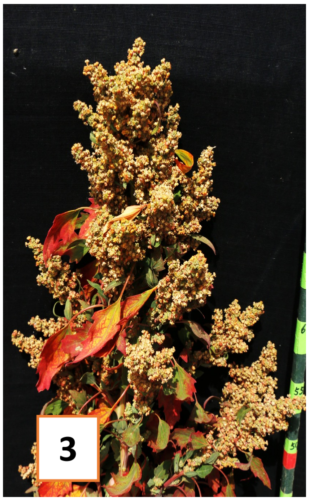

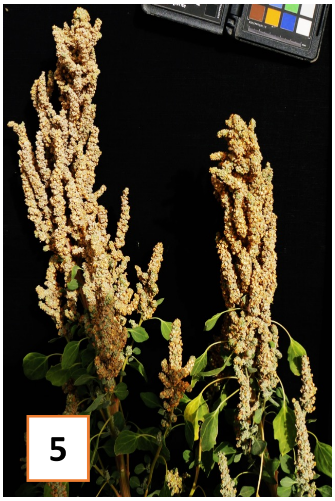

Panicle density

1	3	5	7
Lax (loose)<br><br>Glomerules sparsely spaced, panicle axes easily visible	Intermediate<br><br>Glomerules tighter but with panicle axes still visible	Rarely seeing primary axis	Compact<br><br>Glomerules tightly packed, no panicle axes visible

Panicle density

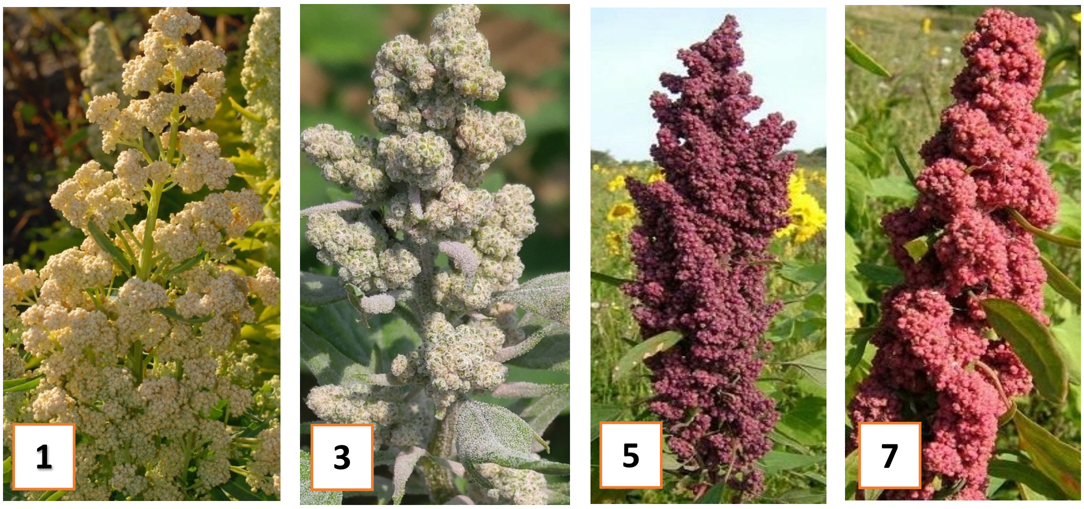

Panicle leafiness

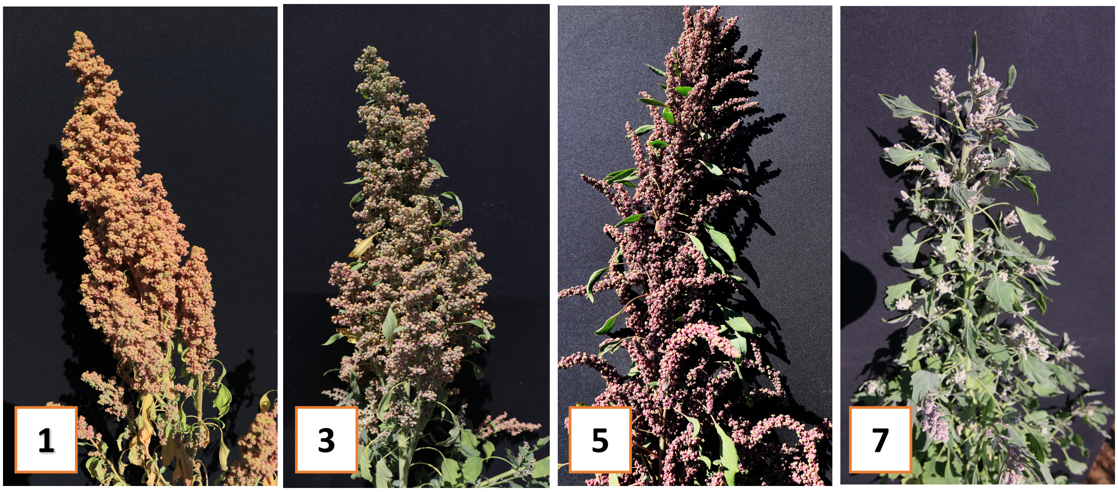

Panicle color

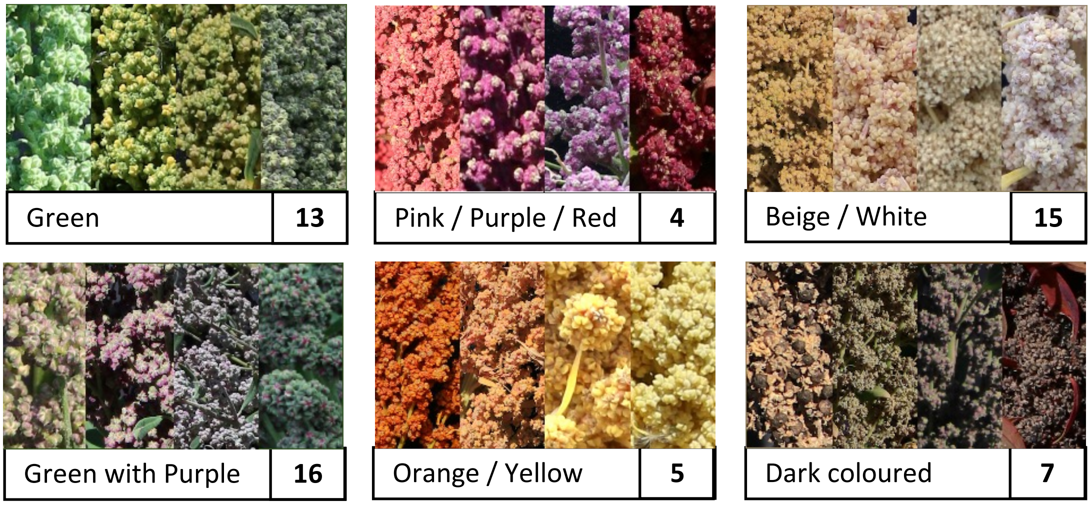

Stem leaf shape

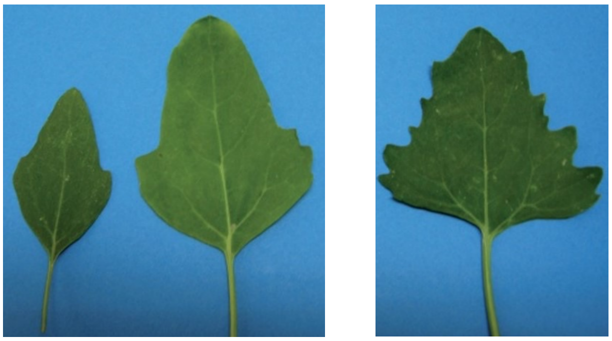

Rhomboidal (1)

Triangular (2)

Stem leaf margin

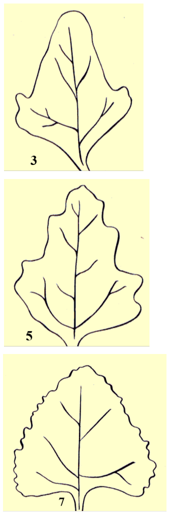

Entire (1)

Dentate (3)

Serrate (5)

Growth stage

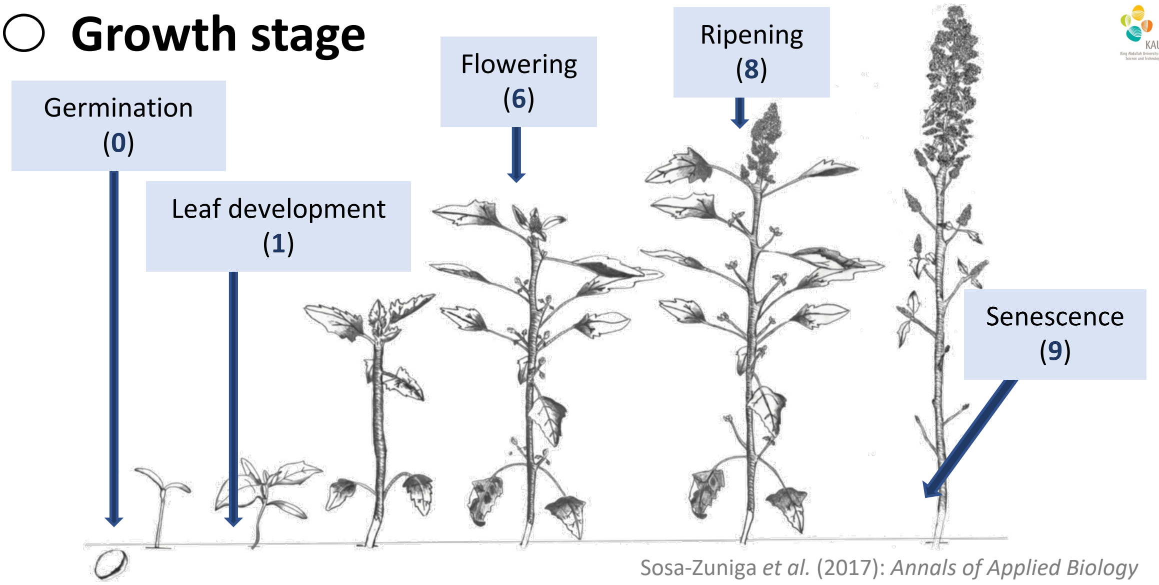

Sosa-Zuniga *et al.* (2017): *Annals of Applied Biology*

BBCH Code Two-digit Description (Sosa-Zuniga et al. 2017)

Principal growth stage 0: germination
00 Dry seed
01 Initiation of seed imbibition
03 Seed imbibition completed
05 Radicle emergence from seed
07 Emergence of hypocotyl
08 Hypocotyl with cotyledons growing towards soil surface
09 Emergence of cotyledons through soil
Principal growth stage 1: leaf development
10 Cotyledons fully emerged
11 First pair of leaves visible
12 Second pair of leaves visible
19 Nine pair of leaves visible. If required, coding can continue following the same scheme.
Principal growth stage 2: formation of side shoots
20 Visible lateral buds or expanded leaves without lateral stems
21 One side shoot visible
22 Two side shoots visible
29 Nine side shoots visible. If required, coding can continue following the same scheme.
Principal growth stage 3: stem elongation (omitted)
Principal growth stage 4: development of harvestable vegetative parts (omitted)

Principal growth stage 5: inflorescence emergence
50 Inflorescence present but still enclosed by leaves
51 Leaves surrounding inflorescence separated, inflorescence is visible from above
59 Inflorescence visible, but all the flowers are still closed
Principal growth stage 6: flowering
60 Beginning of anthesis: main inflorescence flowers with first extruded anthers
67 Early end of anthesis: main inflorescence flowers with first senesced anthers
69 Complete anthesis: main inflorescence flowers with senesced anthers
Principal growth stage 7: fruit development
70 Fruit set: ovary thickening and first visible grains in the main stem
Principal growth stage 8: ripening
81 Milky grain, easily crushed with fingernails, liquid content and green pericarp
85 Thick grain, easily crushed with fingernails, white pasty content, green, beige, red or black pericarp
89 Ripe grain, difficult to crush with fingernails, dry content, the grain has a beige, red or black color on its outside. Ready to harvest.
Principal growth stage 9: senescence
91 Only basal leaves are dry
93 Leaves of the first half portion of the plant, starting from the base, are dead
95 All leaves are dead; stem colour turns from yellow to brown
97 Plant dead and dry
99 Harvested product

BBCH scale examples

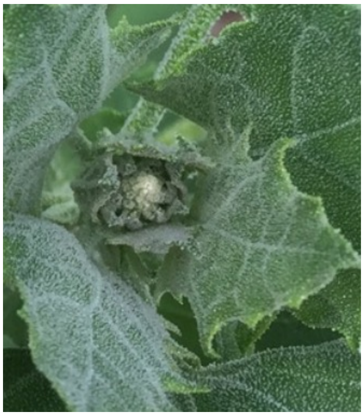
52

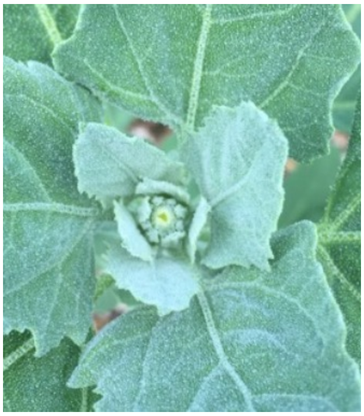
53

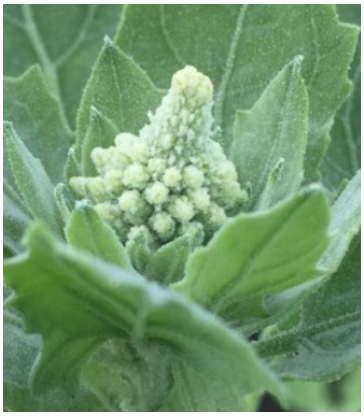
54

BBCH scale examples

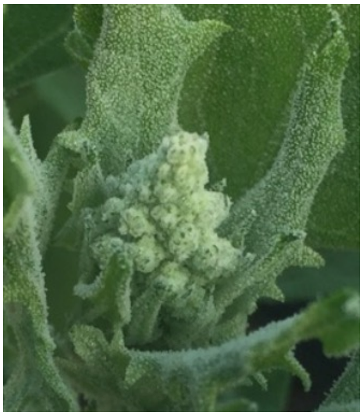

57

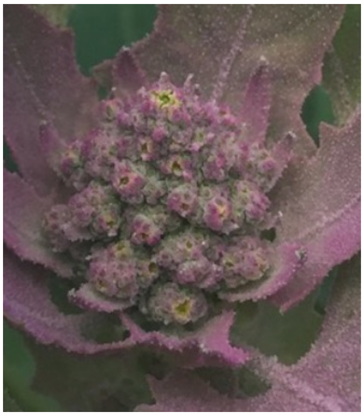

59

- Flowers still enclosed

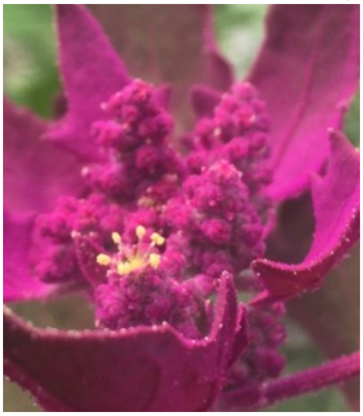

60

- First extruded anthers

BBCH scale examples

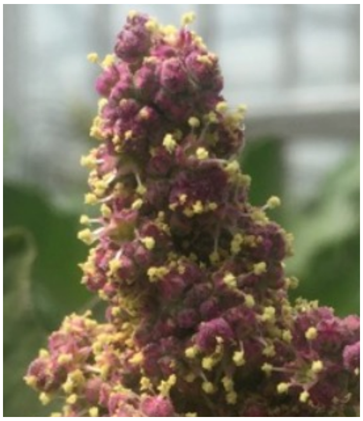

67

- Anther dehiscence

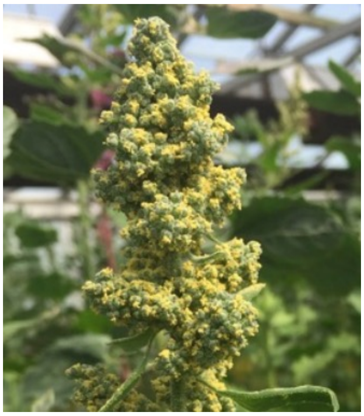

69

- Senesced anthers

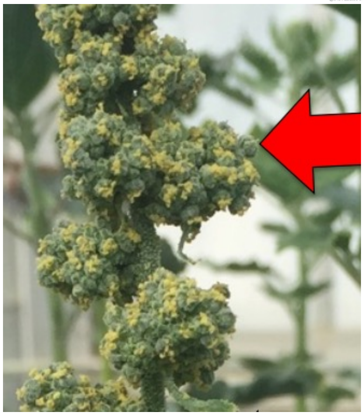

70

– Ovary thickening

Diseases

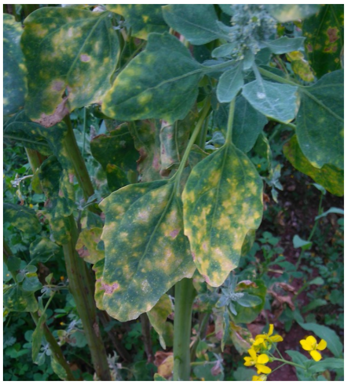

a

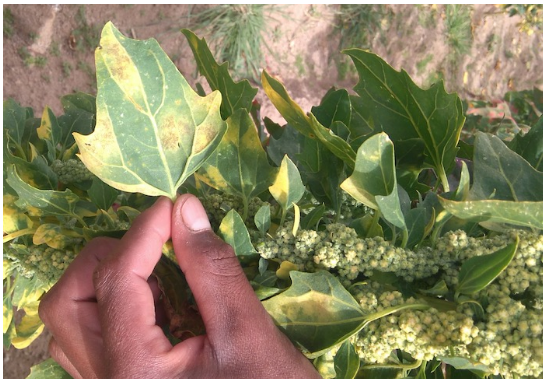

b

a) Leaves of wild-growing *Chenopodium* plants showing symptoms corresponding to the presence of complex pathogen infections.

b) An infected plant with primarily downy mildew disease.

Diseases – lesions on leaf surface

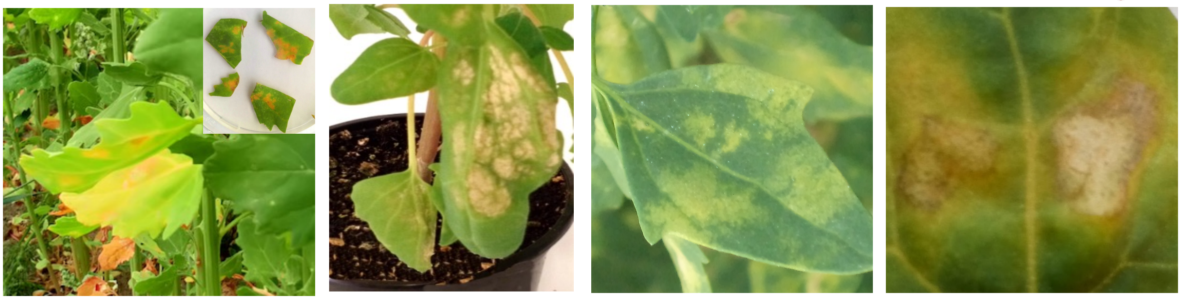

a b c d

Lesions on leaf surface: a) pale or yellow chlorotic lesions with or without a halo and occasional pink-orange discoloration caused by a complex of leaf pathogens, b) bronze irregular lesions caused by *Alternaria* sp., c) diffuse chlorotic spots caused by *P. variabilis* and d) concentric and chlorotic halo under artificial inoculation with *Alternaria* sp.

Diseases – sporulation on abaxial leaf surface

UNIVERSITY OF COPENHAGEN

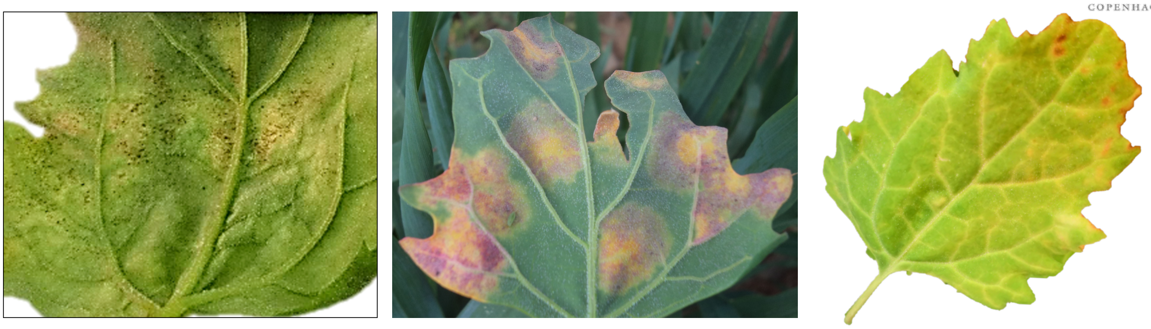

a) Black dots showing downy mildew sporangia

b) Dark grey-violaceous sporulation caused by downy mildew (*Peronospora variabilis*)

c) Vein discoloration, general chlorosis and pink-orange spots caused by *Fusarium* sp. (Colque-Little, 2020)

Diseases – lesion type

UNIVERSITY OF COPENHAGEN

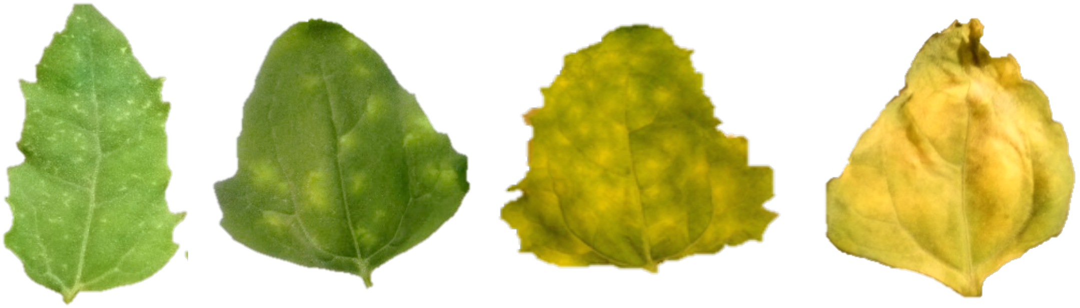

a b c d

Lesion type on upper surfaces and amount of disease ranging from a) dots, b) dots expanding, c) diffuse, and d) extensive (Colque-Little et al, 2021)

Diseases – scoring for area covered

UNIVERSITY OF COPENHAGEN

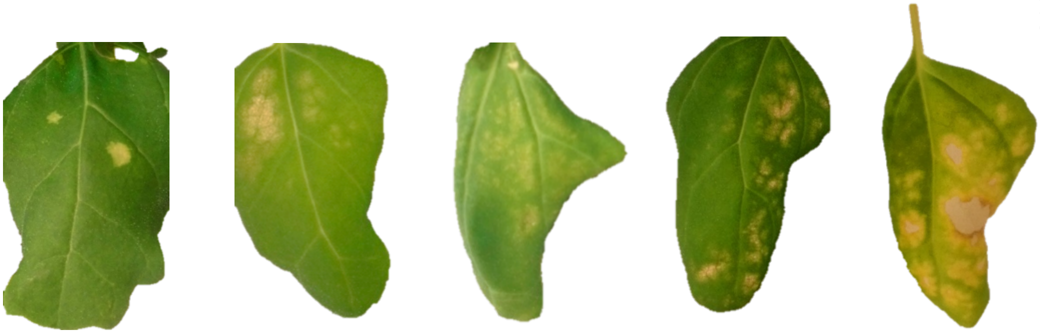

1 = 0%–10% 2 = 11%–25% 3 = 26%–50% 4 = 51%–70% 5 = 71%–100%

Severity phenotyping scale 1-5 used for assessing the percentage of the leaf area affected. Leaf examples given represent different degrees of disease severity during the infection of *Alternaria* sp. on quinoa leaves

Examples for stem diseases

UNIVERSITY OF COPENHAGEN

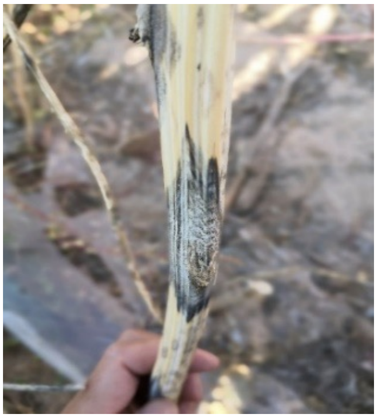

Quinoa black stem caused by *Ascochyta caulina* with the presence of dark structures (pycnidia) (Yin et al., (2020))

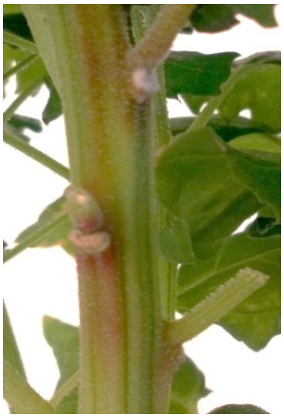

Pink stem and light pink mycelia corresponding to *Fusarium* sp. (Colque-Little et al., (2020))

Examples for panicle diseases

UNIVERSITY OF COPENHAGEN

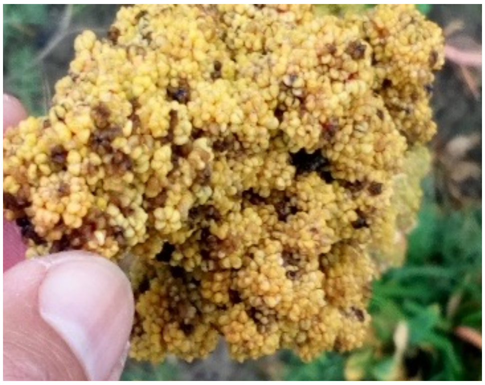

a

A panicle infected with *Alternaria* sp.

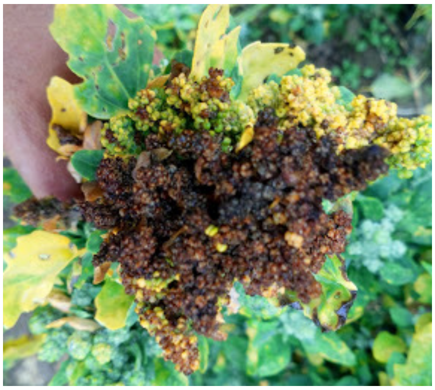

b

A panicle predominantly infected with *Cladosporium* sp. at the end of the season.

(Photos by Colque-Little 2020)
