# Supplementary material for: Quinoa Phenotyping Methodologies: An International Consensus
Source: Plants (Basel). 2021 Aug 24;10(9):1759. doi: 10.3390/plants10091759 (PMC8472428; doi:10.3390/plants10091759)
Supplement: Supplementary file 1 [file plants-10-01759-s001.zip › Supplementary file 2_blue card template.pdf]

Supplementary file 2: Blue card template

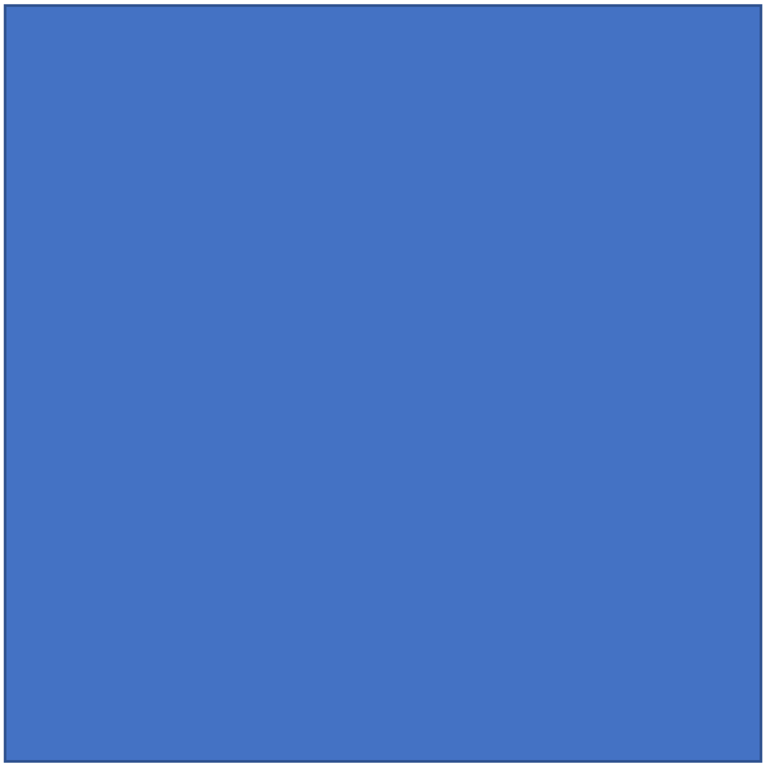

This template for the blue card is in the dimensions needed (10 × 10 cm^2 square) for use of the in-field seed morphology descriptor method (section 9.2.1). It may be printed on a paper of medium to heavy weight and cut out before use.
