# Supplementary material for: Quinoa Phenotyping Methodologies: An International Consensus
Source: Plants (Basel). 2021 Aug 24;10(9):1759. doi: 10.3390/plants10091759 (PMC8472428; doi:10.3390/plants10091759)

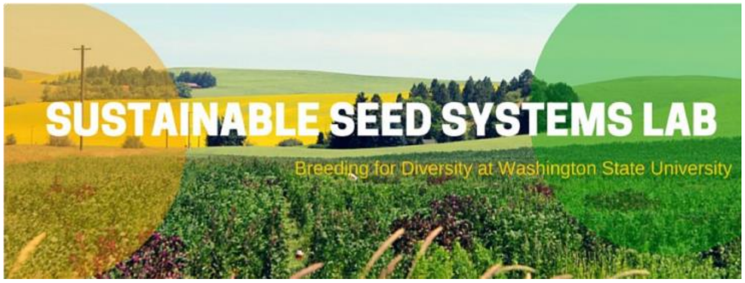

Prepared by Evan Craine (evan.craine@wsu.edu)

- Computer + Monitor
  - You will install a Linux operating system onto this machine
    - Check with Nathan Miller (nathan.miller@wisc.edu) re: correct version to install
  - Dell Optiplex 9010 SFF Computer, Intel Core i5-3470 3.2 GHz, 16 GB RAM, 2 TB HDD, DVD-RW, Windows 10 (Renewed)
- External Hardrive
  - For image backup and storage off of computer
  - Option 1
    - WD 5TB Elements Portable External Hard Drive, USB 3.0 - WDBU6Y0050BBK-WESN
  - Option 2
    - LaCie Rugged Raid Shuttle 8TB External Hard Drive Portable HDD - USB-C USB 3.0 Compatible, Drop Shock Dust Water Resistant, for Mac and PC Computer Desktop Laptop, 1 Mo Adobe CC (STHT8000800)
- Barcode Scanners
  - 1D and 2D for flexibility (QR code = 2D)
  - Handsfree Option (what we use with NIR analyzer)
    - TEEMI Handsfree 1D 2D Barcode Scanner, with Top Trigger Button, QR Omnidirectional Desktop USB Automatic Bar Code Imager, Capture Screen barcodes, TMSL-60 White
  - Wireless with Trigger Mode, Continuous Scan Mode and Auto-Sensing Mode (what we use with scanning system)
    - TEEMI Bluetooth Barcode Scanner with USB Cradle, 1D 2D QR Wireless USB Imager with Vibration Alert for iOS Android Devices, Max 300 Yards Transfer Range, Handsfree Screen Scanning
- **Flatbed Scanners**

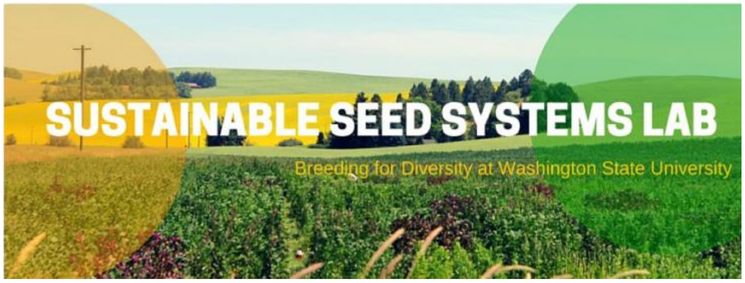

Prepared by Evan Craine (evan.craine@wsu.edu)

- Unit will depend on resolution needs, size, cost, etc.
  - We found that 1200 dpi works well as a balance between resolution and time for image capture
  - The unit below has capability for 4800 dpi optical resolution
- Epson Perfection V39 Color Photo & Document Scanner with Scan-To-Cloud & 4800 Optical Resolution,Black
- https://www.amazon.com/gp/product/B00SSXQ7Q2/ref=ppx_yo_dt_b_asin_title_o03_s00?ie=UTF8&psc=1

- **USB cables to connect scanners**
  - You may need to create additional USB ports on the machine
    - One option
  - USB cable length depends on how far your scanners are from the computer USB ports
    - We purchased 6, 6-ft cables for 6/8 scanner (2/8 scanners had sufficient stock cable lengths)
    - Micro USB Cable Android Charger, JSAUX (2-Pack 6.6FT) Micro USB Android Charger Cable Nylon Braided Cord Compatible with Galaxy S7 S6 J7 Edge Note 5, Kindle. MP3 and More-Red
      - This option also comes in a 3.3ft+6.6ft option and a 6.6ft+10ft option
- **Vacuum**
  - Helpful for removing small seeds from scanners
    - Use a piece of cardboard cut to the scanner width as a “squeegee” to move all the seeds to the bottom edge before vacuuming
  - Cordless option
    - BLACK+DECKER dustbuster Handheld Vacuum, Cordless, 16V (CHV1410L)
    - (battery will be depleted if using for more than 6 hours)

Prepared by Evan Craine (evan.craine@wsu.edu)

- **Seed Corral** 🤠
    - Useful for keeping seeds on scanner in the desired field of view

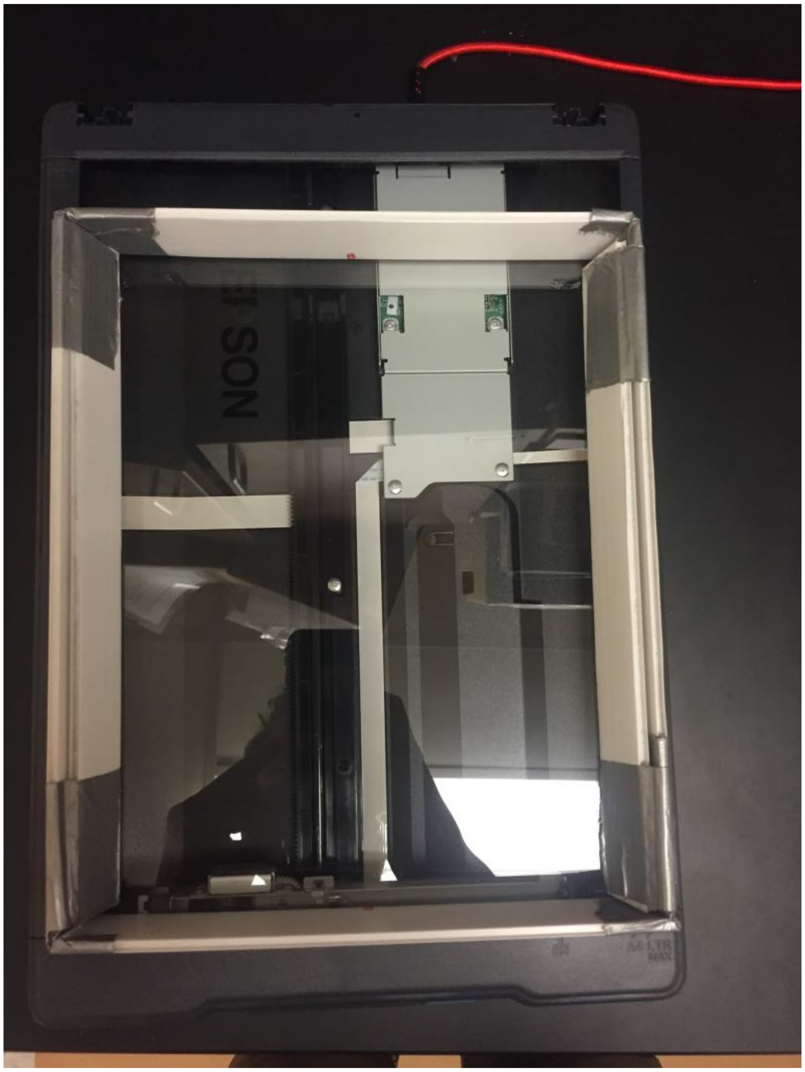

    -
- **Printed sheets to streamline workflow**

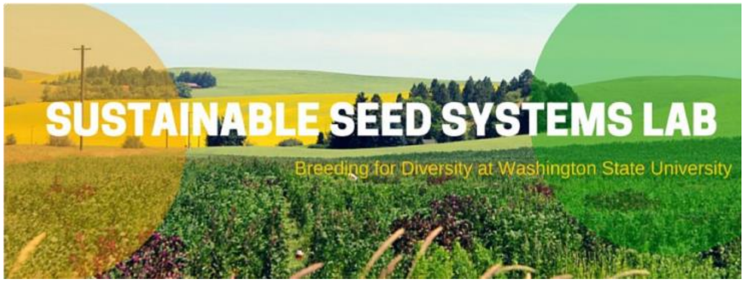

Prepared by Evan Craine (evan.craine@wsu.edu)

- Create QR codes with readable text to scan as needed to assist with the required actions; this will depend on your system and needs
- Set file path for working directory

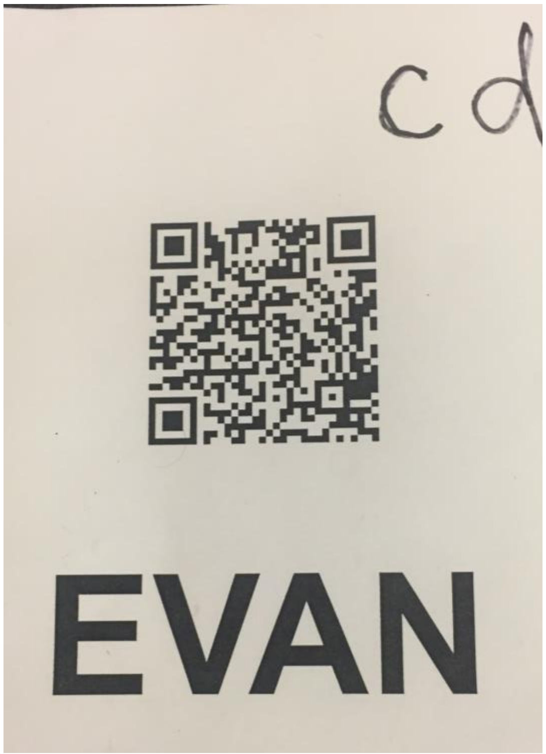

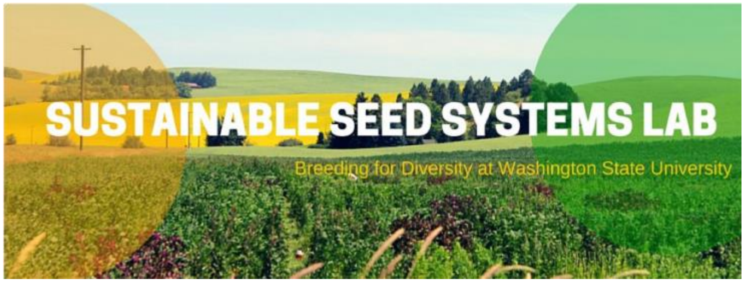

Prepared by Evan Craine (evan.craine@wsu.edu)

- Start command

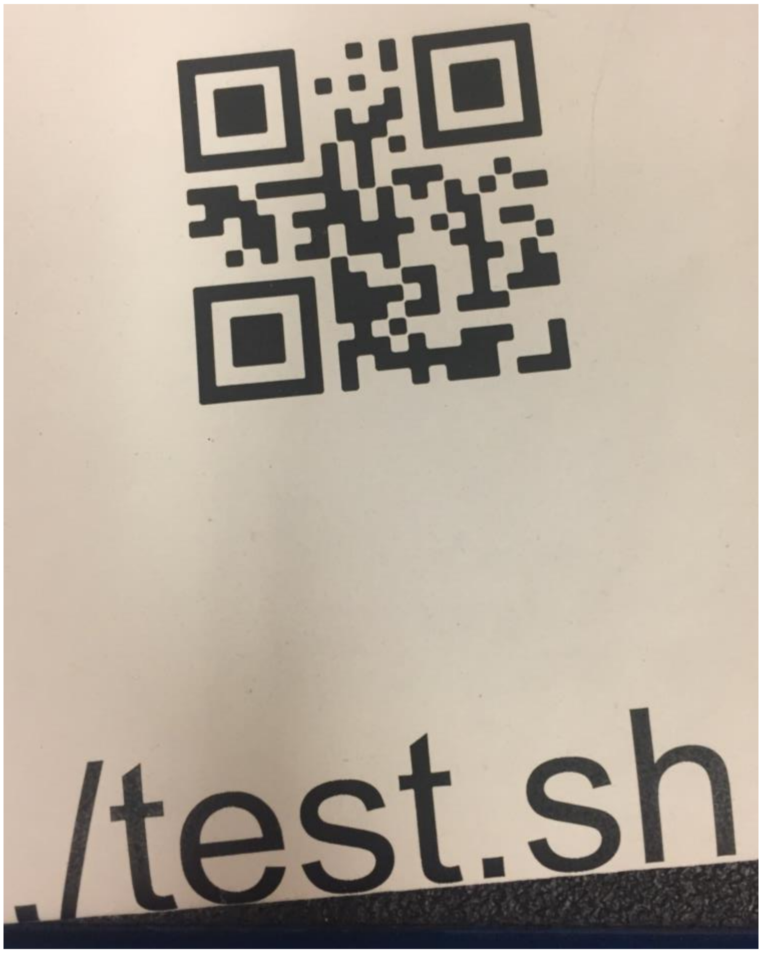

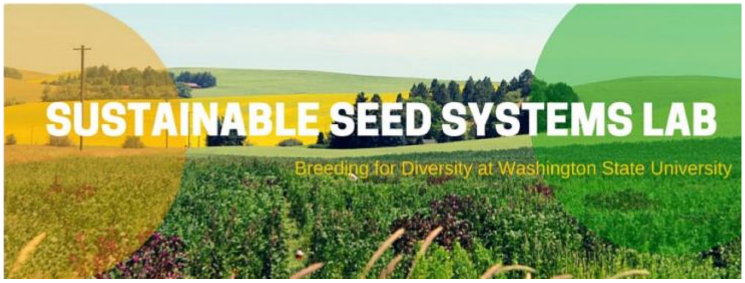

Prepared by Evan Craine (evan.craine@wsu.edu)

- Scanner and corresponding sample ID
    - Ex. scan scanner ID (WHITE) < SPACE < Packet ID < SPACE; repeat with next scanner and sample)
    - This paper is taped to a cardboard box, spray painted matte/flat black inside to create a dark background (you may need a different color background depending on seed colors)
    - We now use a blue background. The underside of the scanner lid, acting as the image background, has been painted using the flat, matte paint colour "Blueberry Festival", which can be characterized by red, green and blue values 73, 139, and 184, respectively and HEX #498BB8 (Valspar Paints, Cleveland, Ohio, U.S.).

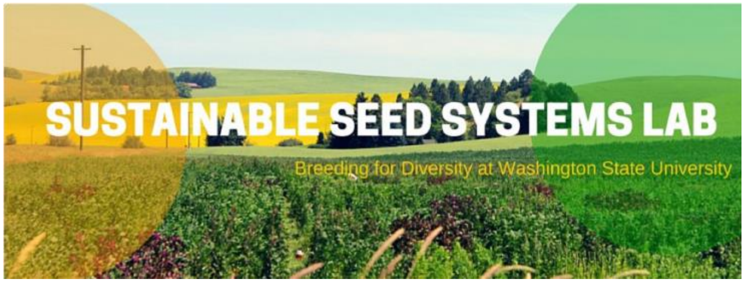

Prepared by Evan Craine (evan.craine@wsu.edu)

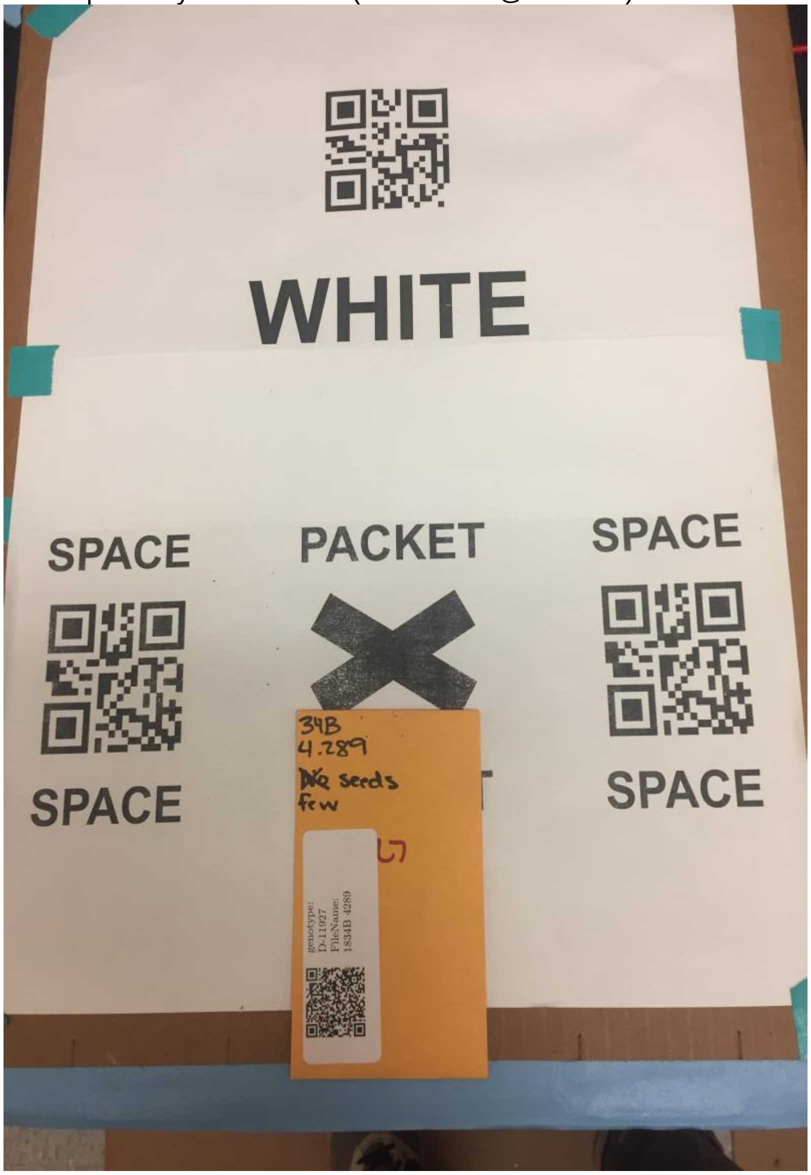

Supplement: Supplementary file 1 [file plants-10-01759-s001.zip › Supplementary file 3_Equipment List.pdf]
