# Supplementary material for: Quinoa Phenotyping Methodologies: An International Consensus
Source: Plants (Basel). 2021 Aug 24;10(9):1759. doi: 10.3390/plants10091759 (PMC8472428; doi:10.3390/plants10091759)
Supplement: Supplementary file 1 [file plants-10-01759-s001.zip › Supplementary file 4_QuinoaCollab_NIR_Scan.pdf]

Washington State University Nutritional Phenotyping Laboratory

Sample Organization and Tracking

Sample organization and tracking is maintained throughout the phenotyping pipeline using a system that relies on 2D digital barcodes (i.e. QR codes), barcode scanners, and USB drivers. The system is designed for quality assurance and quality control, by automating sample data entry and processing.

The first stage of this process requires generating sheets of barcode labels from seed collection packing lists provided by collaborators (Figure 1). An inventory is taken by matching QR labels with the corresponding seed packet. Each affixed label contains a QR code embedded with the sample information, along with space dedicated to human-readable text. Unique sample identifiers originating from each individual seed colelction's identification scheme comprise the embedded information. The most useful information is selected and printed as human-readable text. A crucial field to include, usually embedded in the barcode, is a unique filename that can be used to query datasheets. This allows for data from each phenotyping station (NIR, subsampling, seed scanning, XRF) to be compiled.

Figure 1. Example sheet of QR code (i.e. 2D barcode) labels featuring the machine readable and human readable information. A PDF of QR code sheets is printed onto Avery 5620, or similar, labels. Credit – Evan Craine

genotype: BO-55 FileName: 187B 1001	genotype: Moroccan Yellow FileName: 187B 1002	genotype: Bouchane-3 FileName: 187B 1003
genotype: PI-614889 FileName: 187B 1004	genotype: ICBA-Q5 FileName: 187B 1005	genotype: PI-614927 FileName: 187B 1006
genotype: D-12225 FileName: 187B 1007	genotype: PI-665276 FileName: 187B 1008	genotype: CHEN-298 FileName: 187B 1009
genotype: D-12196 FileName: 187B 1010	genotype: D-11980 FileName: 187B 1011	genotype: CHEN-465 FileName: 187B 1012

NIR Analyzer – Data Collection

A DA7250 Near Infrared Analyzer (PerkinElmer Instruments, Springfield, Illinois, United States) is used to generate seed composition data for quinoa nutritional parameters (Figure 2). Whole, raw quinoa seed is analyzed. Should seed samples contain any impurities (stems, rocks, dirt, etc.) that could bias the spectral signature of the sample, the sample is cleaned by passing the seed through metal screens to remove bulk material, followed by the removal of fine material using a Holland 4110.23.00 seed blower. The

sample is placed in an appropriately sized sample cup, depending on total sample volume available for analysis, and leveled using a “Z” pattern to ensure consistent sample preparation between samples (Figure 3). Data is reported as an average of two repacks, to provide a more representative analysis of each sample. A repack consists of emptying the sample cup into the sample preparation tray, pouring the seed back before preparing as described above.

Figure 2. Left to right: sample preparation tray and ergonomic sample leveler; cups of various sizes to accommodate a wide range of sample volumes; TEEMI 1D & 2D USB Automatic Hands-free desktop barcode scanner; PerkenElmer (formerly Perten Instruments) DA 7250™ NIR Analyzer. Credit – Evan Craine

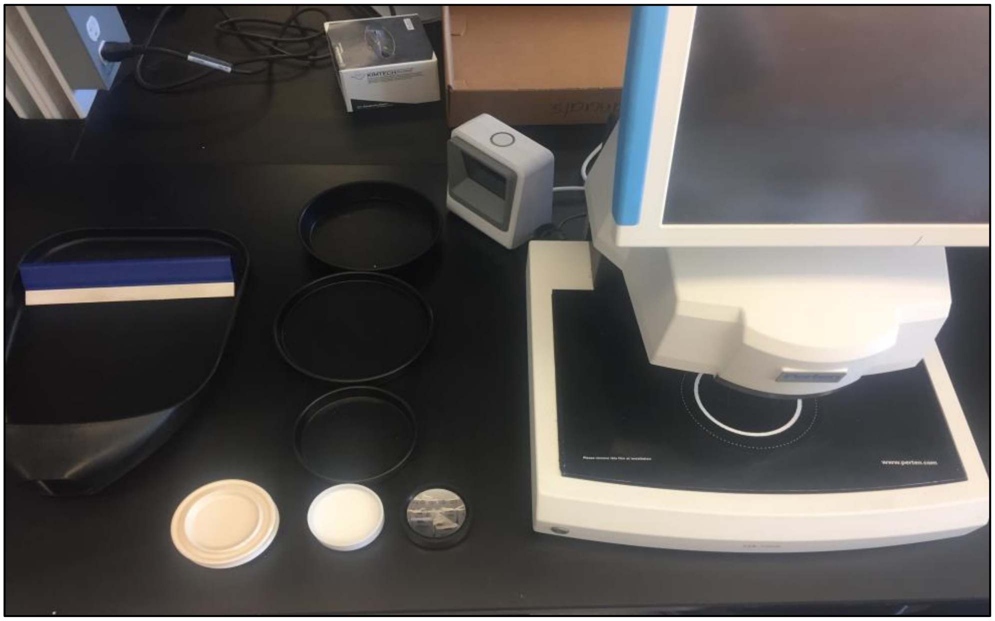

Figure 3. Small “mirror cup” used for samples of ~10g. Light is reflected off of the sample surface and collected to generate spectral data from 950-1650 nm, at 5 nm increments. Credit – Evan Craine

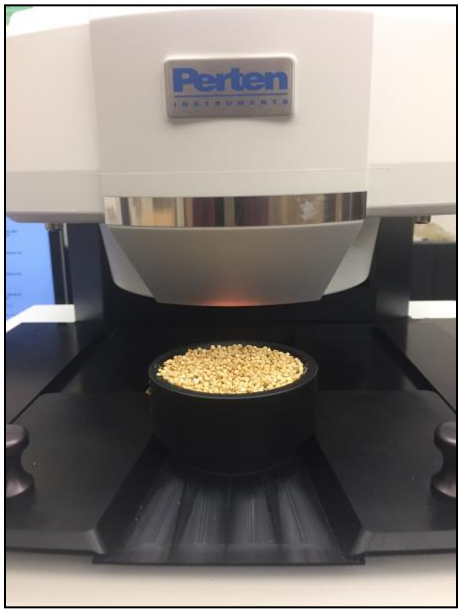

NIR Analyzer – Calibration Development
Stock calibrations did not include amino acid calibrations for quinoa, which have since been added and improved upon. Moreover, the stock calibration was not robust, and poorly predicted novel quinoa samples; NIR analysis is best at interpolation rather than extrapolation. According to PerkinElmer (formerly Perten Instruments), the Nutritional Phenotyping Laboratory at WSU is the only group working on developing improved quinoa calibrations using the DA 7250 ™. Two improved versions of the stock calibration have been developed, with the most recent version (V3) used to generate the predicted values for the 2018 seed collections. Calibration metrics are provided in Appendix II – Table 1.

The stock calibration was developed using 27 samples provided by a group in Sweden (Figure 4 – white data points; PerkinElmer Instruments pers. comm.). The second version (V2) added samples from WSU breeding program materials and field research trials (Figure 4-colored data points). These samples were randomly selected across a normal distribution of crude protein content predicted using the stock calibration, and the Agricultural Experiment Station Chemical Laboratories (AESCL) at the University of Missouri-Columbia performed "wet chemistry" analysis of raw quinoa samples to generate reference data.

Figure 4. Example of regression data for predicted (i.e. NIR) and observed (i.e. wet chemistry) crude protein content in Quinoa Calibration (V2). Calibration metrics are presented in the top left legend of the figure. RMSECV = root mean square error of cross validation (CV); SECV = standard error of CV; RPDCV = ratio of performance to deviation CV (the ratio between the standard deviation of a variable and the standard error of prediction of that variable by a given model). Credit – Perkin Elmer

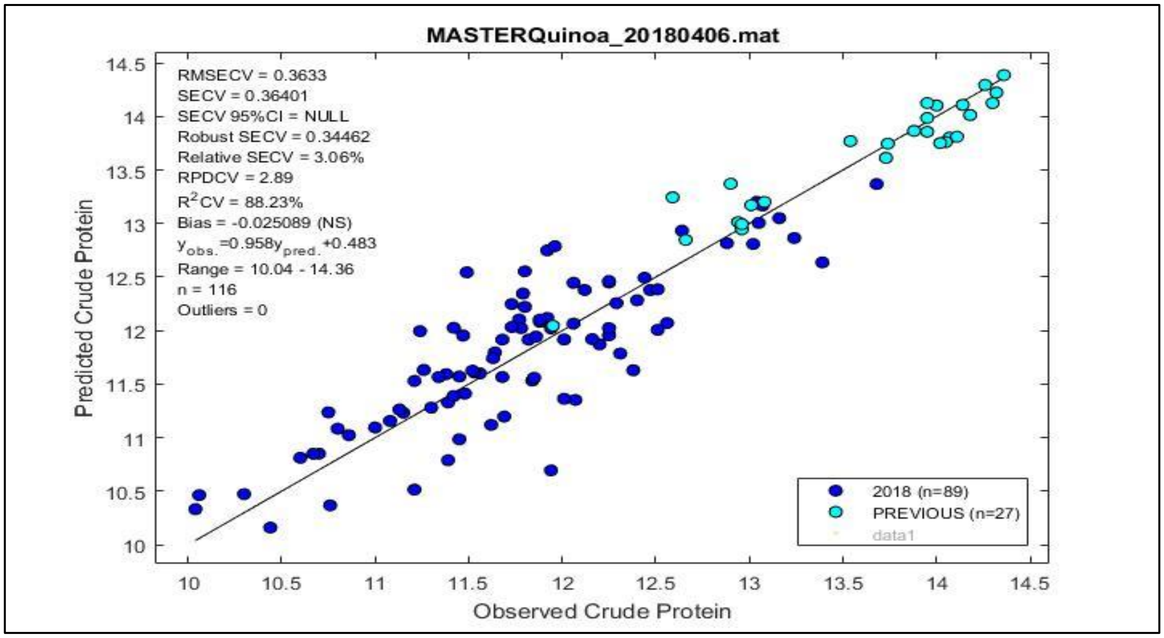

The current NIR calibration, V3, incorporated 37 samples selected from the 2018 NACRA (i.e. 18KA) seed collection. This is the only seed collection that had sufficient sample amounts for wet chemistry analysis (minimum of 25 grams). Scatter plots of predicted and observed values (i.e. reference values), in addition to calibration metrics, for each parameter are provided in Appendix III. These data are provided by David Honigs, who also developed V3 of the NIR calibration (PerkinElmer).

PerkinElmer conducted sample selection using the Kennard-Stone Method on spectral data. Reference data was again generated by the AESCL; however, 10 blind duplicates were included to measure and account for laboratory standard error in the calibration. Following calibration development, PerkinElmer performed 8 fold cross validation in triplicate to measure calibration prediction accuracy metrics, which are reported as an average measure. These data, referred to as calibration metrics, are presented in Appendix II and III. Perten Instruments repredicted all the NIR data for the 2018 seed collections using V3 of the calibration. Mahalanobis distance (MD) and nearest neighbor distance (NND) were calculated for each parameter. Outliers can be identified using the MD values from the reference samples and represent samples that are outside the reference data range or poorly fit the model. Parameters are reported on an as is basis (gram $100g^{-1}$ sample), unless otherwise noted. Crude protein content is calculated as percent nitrogen multiplied by 6.25 (LECO). It is worth noting that prediction of hydroxylysine and hydroxyproline is extremely limited by the finite reference data range, and this is also true to some extent for tryptophan. Lanthionine and orthinine are not included in the calibration because of a lack of useful reference data (i.e. values reported as 0.00).

Seed Scanning System – Data Collection
The Nutritional Phenotyping Laboratory removes a small subsample (approximately 1-2 grams) of each sample for seed scanning. The weight of each subsample is measured using an Ohaus Scout SPX123 Analytical Balance (Parsippany, New Jersey, United States), with a capacity of 120 grams and measurement to 0.001 grams. A barcode scanner is used to populate the sample information into an Excel spreadsheet, and USB driver is used to facilitate transfer of the balance output directly to the spreadsheet. Seed scanning is carried out using flatbed scanners (Epson Perfection V39; 1200 dpi), and provides several useful measurements of the seed phenotype. For example, seed size, seed color, and seed shape can be quantified. The number of seeds in each subsample is automatically counted, and this information is combined with the weight of the subsample to calculate the 1000 seed weight. Using data from the 2018 Germany seed collection, hand-counted 1000 seed weight was compared to scanner-counted 1,000 seed weight. The two methods have a significant, strong postitive correlation ($R = 0.95$, $p < 2.2e-16$) (Figure 6). The “Arabidopsis Seed Method” in the “Phytomorph Image Phenomics Tool Kit” is used for image analysis (Figure 5 (A)). However, this method has been replaced by a new algorithm incorporating a blue background (Figure 5 (B)). This work is performed in collaboration with Nathan Miller at the University of Wisconsin-Madison and Yang Hu at Washington State University. Nathan Miller developed the image analysis software, and Yang Hu adapted the Python shell scripts shared by UW-

Madison to support simultaneous operation of up to 8 flatbed scanners and automated hierarchical file storage based on QR code information

Figure 5. (A) Oro de Valle seed scan mask image using the Arabidopsis Seed Method. Green colored seeds are touching and identified as a larger object relative to the smaller objects (i.e. individual seeds colored red). Larger objects are dived by the average smaller object size to determine the number of smaller objects present in the larger objects, and their characteristics. (B) Scan image of Titicaca seed (2019 Australia seed collection) using blue background. Image resolution reduced from 1200 DPI to 250 DPI. Objects, such as fine debris, that are smaller than the seed objects are filtered out and excluded from the image analysis. Image Credit – Nathan Miller

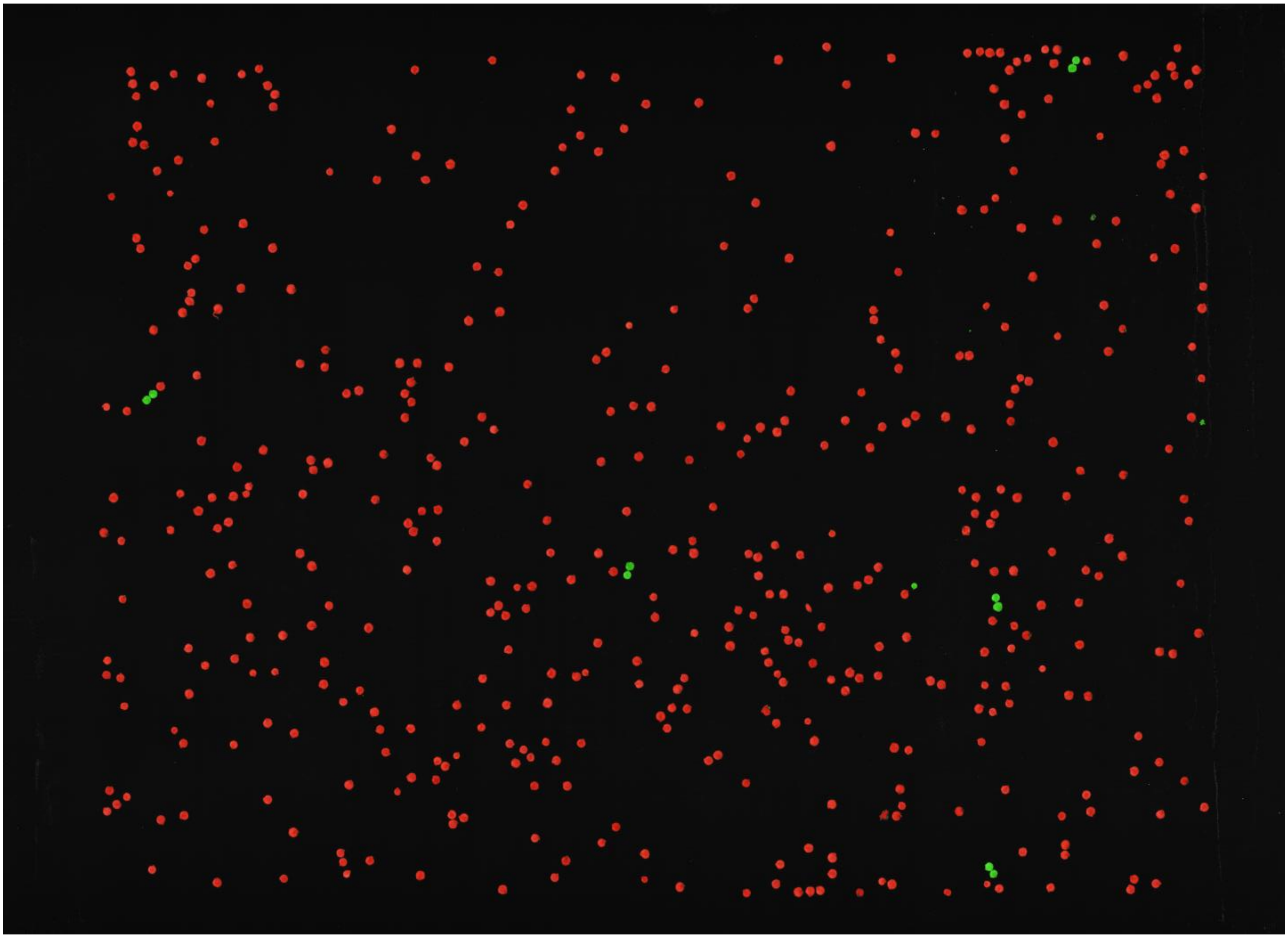

(A)

(B)

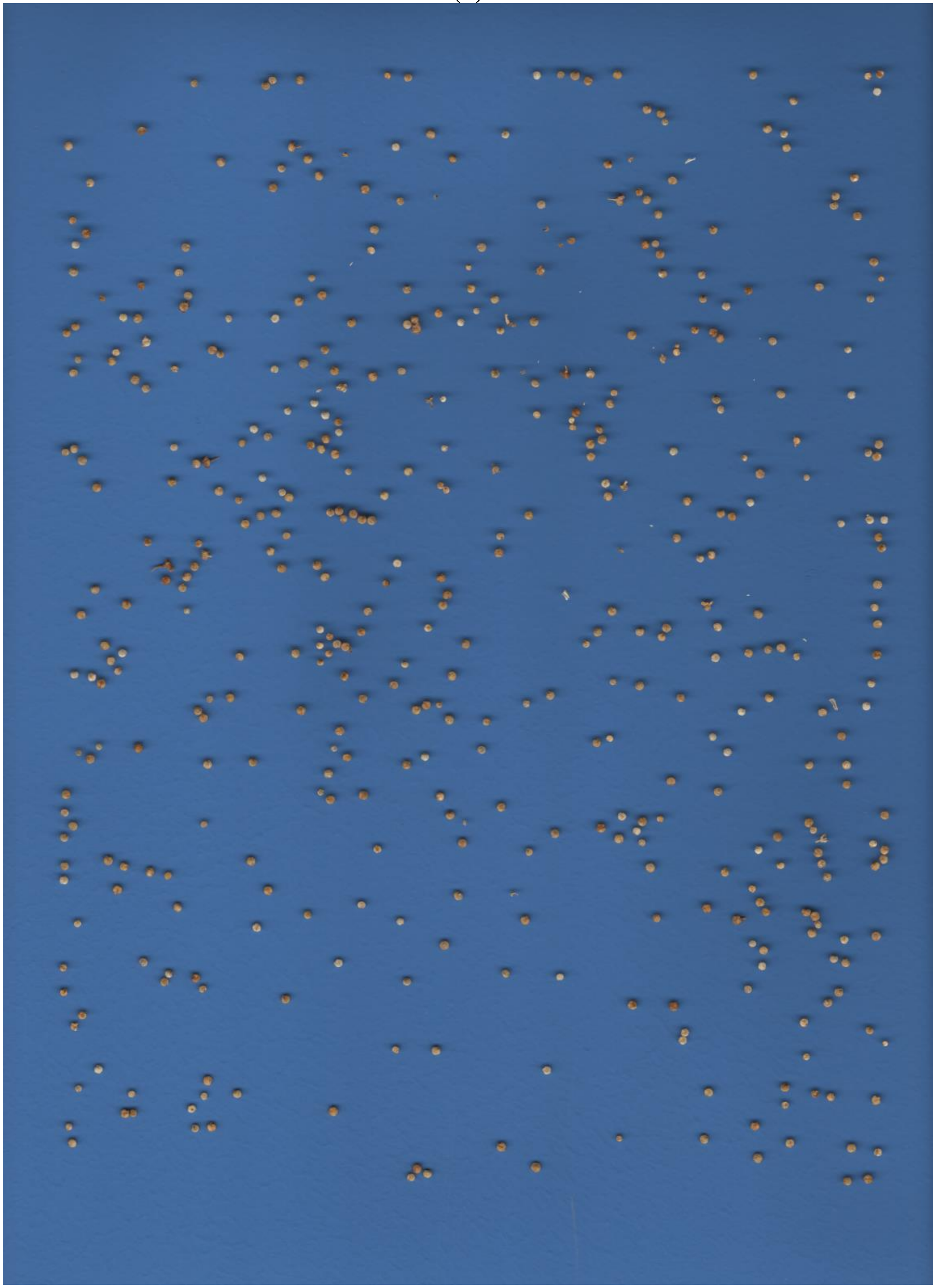

Figure 6. Hand-counted 1000 seed weight (x-axis; 2018 (manual)) compared to scanner-counted 1,000 seed weight (y-axis; 2018 (evan)) for samples from the 2018 Germany seed collection. Image Credit – Dilan Sarange

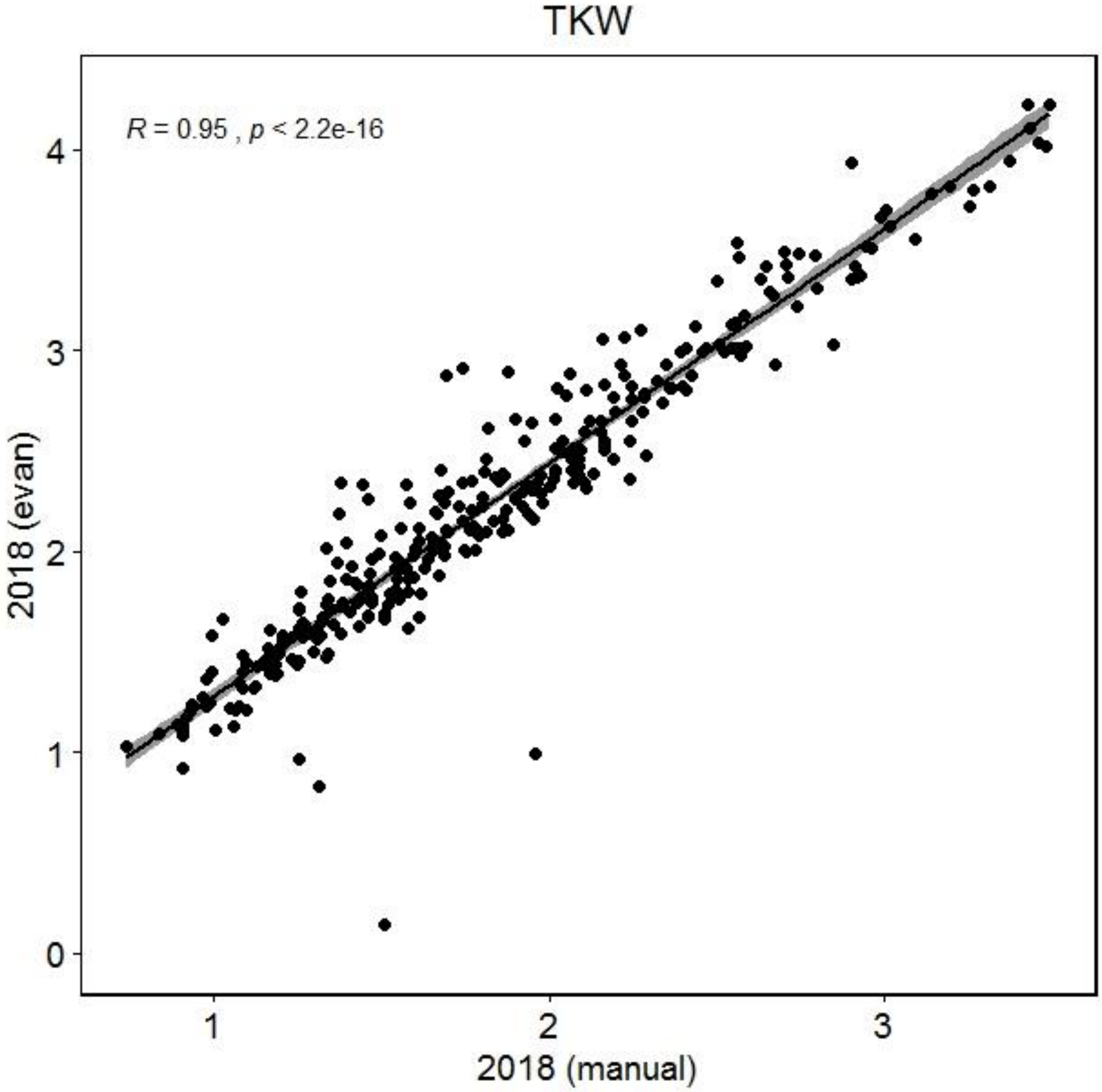

Seed Scanning System – Metadata for Scan Output File

The "Arabidopsis Seed Method" in the "Phytomorph Image Phenomics Tool Kit" generates numerous parameters useful for characterizing seed phenotypes, and understanding various within and among seeds in a sample. These parameters are listed below. A "default_" prefixes denotes a parameter automatically generated through the analysis, while an "adj_" prefix represents a unit conversion from the "default_" parameter (e.g. dpi to mm).

<u>default_AverageArea</u>
Average area calculated as a mean of all seed areas
adj_AverageArea_mm2
The average area of all seeds, adjusted to mm^2
default_StdArea
Standard deviation of the average area
adj_StdArea_mm2
Standard deviation of the average area, adjusted to mm^2

default_MajorAxisLength
Length of the major axis, calculated as a mean of all seed major axis lengths
adj_MajorAxisLength_mm
Average major axis length, adjusted to mm
default_StdMajorAxisLength
Standard deviation of the average major axis length
adj_StdMajorAxisLength_mm
Standard deviation of the average major axis length, adjusted to mm

default_MinorAxisLength
Length of the minor axis, calculated as a mean of all seed minor axis lengths
adj_MinorAxisLength_mm
Average minor axis length, adjusted to mm
default_StdMinorAxisLength
Standard deviation of the average minor axis length
adj_StdMinorAxisLength_mm
Standard deviation of the average minor axis length, adjusted to mm

default_AverageEccentricity
Average eccentricity calculated as a mean of all seed eccentricities. A circle has an eccentricity of 0. Calculated as $\sqrt{1-\frac{b^2}{a^2}}$ where a = length of the semi-major axis (half of the major axis) and b = length of the semi-minor axis (half of the minor axis).
default_StdEccentricity
Standard deviation of the average eccentricity

For the following, the second operation is listed first, followed by the first operation

default_AverageMeanRed
Average of the mean red pixels
Mean of the seed red pixels for each seed, then average of the red pixel means for all seeds
default_StdMeanRed
Standard deviation of the red pixel means
Mean of the seed red pixels for each seed, then standard deviation of the red pixel means for all seeds
default_AverageMeanGreen
Average of the mean green pixels
Mean of the seed green pixels for each seed, then average of the green pixel means for all seeds
default_StdMeanGreen
Standard deviation of the green pixel means
Mean of the seed green pixels for each seed, then standard deviation of the green pixel means for all seeds

default_AverageMeanBlue
Average of the mean blue pixels
Mean of the seed blue pixels for each seed, then average of the blue pixel means for all seeds
default_StdMeanBlue
Standard deviation of the blue pixel means
Mean of the seed blue pixels for each seed, then standard deviation of the blue pixel means for all seeds
default_AverageStdRed
Average of the standard deviations
Standard deviation of the red pixels for each seed, then average of the red pixel standard deviations
default_StdStdRed
Standard deviation of the standard deviations
Standard deviation of the red pixels for each seed, then standard deviation of the red pixel standard deviations
default_AverageStdGreen
Average of the standard deviations
Standard deviation of the green pixels for each seed, then average of the green pixel standard deviations
default_StdStdGreen
Standard deviation of the standard deviations
Standard deviation of the green pixels for each seed, then standard deviation of the green pixel standard deviations
default_AverageStdBlue
Average of the standard deviations
Standard deviation of the blue pixels for each seed, then average of the blue pixel standard deviations

default_StdStdBlue
Standard deviation of the standard deviations
Standard deviation of the blue pixels for each seed, then standard deviation of the blue pixel standard deviations
default_totalCount
Total number of seeds counted in the scanned image
color
AverageMeanRed, AverageMeanGreen, and AverageMeanBlue values used to shade Excel cell using the Excel module script in Appendix I with Visual Basic Editor
SS_g
Subsample weight in grams for the seeds in the scanned image, measured before the seeds are placed on the scanner
1Kseedweight
Calculated as (SS_g/default_totalCount) * 1000. See "18KA_1000SeedWeight" Excel Workbook for predicted 1Kseedweight using seed scanning system to hand counted 1Kseedweight.

Appendix I - Color function to add as macro in Excel to fill cell with color, based on red (R_, green (G) and blue (B) values

```
Function myRGB(r, g, b)

    Dim clr As Long, src As Range, sht As String, f, v

    If IsEmpty(r) Or IsEmpty(g) Or IsEmpty(b) Then
        clr = vbWhite
    Else
        clr = RGB(r, g, b)
    End If

    Set src = Application.ThisCell
    sht = src.Parent.Name

    f = "Changeit(""" & sht & """,""" & _
              src.Address(False, False) & """," & clr & ")"
    src.Parent.Evaluate f
    myRGB = ""
End Function

Sub ChangeIt(sht, c, clr As Long)
    ThisWorkbook.Sheets(sht).Range(c).Interior.Color = clr
End Sub
```

Appendix II – NIR Calibration Metrics and Comparison

Table 1. Calibration metrics presented for V2 and V3 (current) of the whole, raw (unprocessed) quinoa seed analysis profile. The range of values for the reference samples are provided and compared to the values reported by Escuredo et al. (2014) (STATS FROM NIR PAPER). Parameters with a range less than the value reported by Escuredo et al. (2014) have the potential to be improved by incorporating samples into the calibration that increase the range of reference values. This is summarized on the right side of the table, with particular paramters presented in bold text if the V3 range is less than the range reported by Escuredo et al. (2014). Amino acids are reported as g/100g protein. Hydroxylysine and hydroxyproline are poorly predicted. Crude protein, ash, crude fat, moisture and total amino acids (TotalAA) are reported as g/100g sample.

	STATS FROM NIR PAPER (g 100g-1 protein)			STATS FROM WSU CALIBRATION V2 DATA (g 100g-1 protein)					STATS FROM WSU CALIBRATION V3 DATA (g 100g-1 protein)											
Parameter	Min	Max	Range	SECV	R2CV	Min	Max	Range	Range	Min	Max	RMSECV	SECV	Robust SECV	RPDCV	R2CV	MD warning	MD error limit	Range>Paper?	Parameter
Alanine	1.26	3.1	1.84	0.024	0.437	3.84	4.88	1.04	1.99	2.89	4.88	0.022	0.022	0.018	3.036	0.892	6.657	8.173	YES	Alanine
Arginine	1.32	5.69	4.37	0.04	0.735	6.35	8.39	2.04	4.68	4.58	9.25	0.053	0.053	0.044	4.308	0.946	6.565	8.722	YES	Arginine
Aspartic acid	1.49	7.32	5.83	0.04	0.616	7.13	8.73	1.6	3.22	5.51	8.73	0.039	0.040	0.036	3.768	0.930	6.754	8.528	no	Aspartic acid
Cysteine	0.05	0.33	0.28	0.012	0.426	1.56	2.07	0.51	0.76	1.31	2.07	0.010	0.010	0.010	3.188	0.902	6.436	8.359	YES	Cysteine
Glutamic acid	4.32	13.26	8.94	0.084	0.584	10.86	14.98	4.12	7.04	8.22	15.26	0.093	0.093	0.086	3.802	0.931	6.373	8.239	no	Glutamic acid
Glycine	2.03	4.22	2.19	0.028	0.48	5.07	6.11	1.04	1.33	4.78	6.11	0.041	0.041	0.036	2.447	0.834	3.691	5.408	no	Glycine
Histidine	0.63	3.08	2.45	0.015	0.67	2.29	2.91	0.62	1.05	1.96	3.01	0.015	0.015	0.014	4.564	0.952	6.314	7.869	no	Histidine
Isoleucine	0.25	1.43	1.18	0.02	0.652	3.55	4.41	0.86	1.51	2.89	4.41	0.021	0.022	0.019	3.392	0.913	6.586	8.557	YES	Isoleucine
Leucine	0.36	3.51	3.15	0.033	0.579	5.63	6.85	1.22	2.55	4.3	6.85	0.031	0.032	0.029	3.473	0.917	6.361	8.657	no	Leucine
Lysine	1.32	3.66	2.34	0.03	0.528	5.05	6.59	1.54	3.14	3.45	6.59	0.029	0.029	0.033	3.290	0.908	5.875	7.535	YES	Lysine
Methionine	0.05	4.48	4.43	0.013	0.543	1.8	2.46	0.66	1.15	1.31	2.46	0.012	0.012	0.009	2.955	0.886	6.550	8.709	no	Methionine
Phenylalanine	0.68	2.2	1.52	0.02	0.609	3.52	4.28	0.76	1.57	2.71	4.28	0.019	0.019	0.018	3.889	0.934	6.751	8.867	YES	Phenylalanine
Proline	0.84	2.74	1.9	0.025	0.567	3.35	4.48	1.13	1.68	2.80	4.48	0.023	0.023	0.018	2.556	0.847	5.958	8.067	no	Proline
Serine	0.68	3.15	2.47	0.021	0.422	3.36	4.28	0.92	1.39	2.89	4.28	0.019	0.019	0.016	3.176	0.901	6.286	8.050	no	Serine
Taurine			n/a	0.014	0.606	0.9	1.79	0.89	1.96	0.82	2.79	0.012	0.012	0.009	1.669	0.645	6.235	8.190	n/a	Taurine
Threonine	0.28	18.94	18.66	0.018	0.485	3.18	4.02	0.84	1.60	2.43	4.02	0.017	0.017	0.016	3.015	0.890	6.331	7.940	no	Threonine
Tryptophan	0.69	1.71	1.02	0.015	0.269	0.68	1.48	0.8	0.93	0.55	1.48	0.012	0.012	0.009	1.681	0.647	5.504	7.767	no	Tryptophan
Tyrosine	0.53	1.93	1.4	0.016	0.503	2.43	3.05	0.62	0.93	2.12	3.05	0.014	0.014	0.013	3.393	0.913	6.247	8.383	no	Tyrosine
Valine	0.16	2.03	1.87	0.024	0.576	4.31	5.2	0.89	1.84	3.36	5.20	0.024	0.024	0.023	3.260	0.906	6.333	8.687	no	Valine
Hydroxylysine			n/a						0.18	0.05	0.23	0.004	0.004	0.003	1.591	0.605	5.915	7.726		Hydroxylysine
Hydroxyproline			n/a						0.93	0.29	1.21	0.010	0.010	0.011	1.821	0.699	5.815	7.732		Hydroxyproline
Crude Protein			n/a	0.369	0.88	10.04	15.05	5.01	11.95	6.82	18.77	0.394	0.395	0.406	5.521	0.967	6.401	8.599		Crude Protein
Ash			n/a	0.159	0.88	2.15	5	2.85	3.32	2.21	5.53	0.154	0.154	0.129	3.084	0.895	5.847	7.922		Ash
Crude Fat			n/a	0.195	0.864	1.79	4.76	2.97	6.95	0.00	6.95	0.310	0.311	0.316	3.883	0.934	6.515	8.217		Crude Fat
Moisture			n/a	0.237	0.95	6.41	10.35	3.94	3.76	6.41	10.17	0.183	0.183	0.159	6.579	0.977	5.099	6.832		Moisture
TotalAA			n/a						10.06	5.84	15.90	0.413	0.413	0.328	4.018	0.938	6.385	7.963		TotalAA

Appendix III - Scatter plots of predicted (V3 NIR calibration) and observed values (i.e. reference values), in addition to calibration metrics, for each parameter. Image Credit – David Honigs (PerkinElmer)

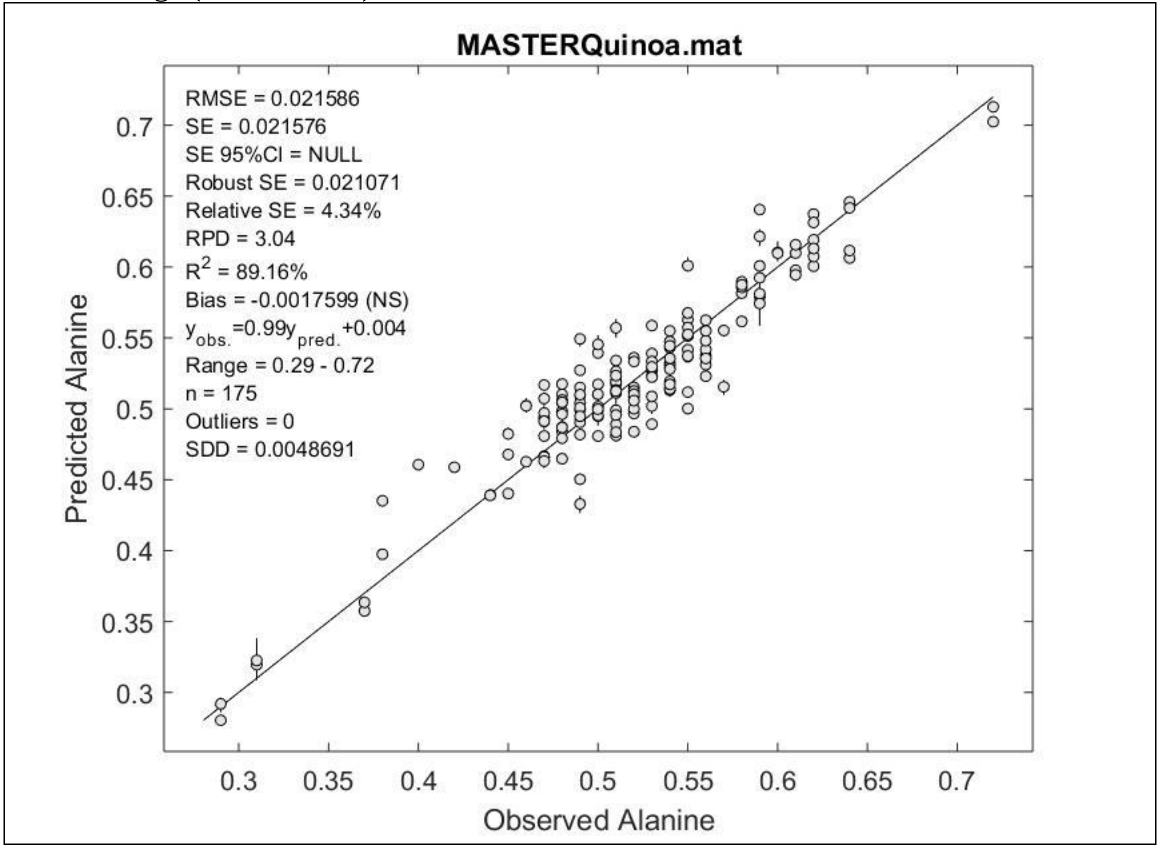

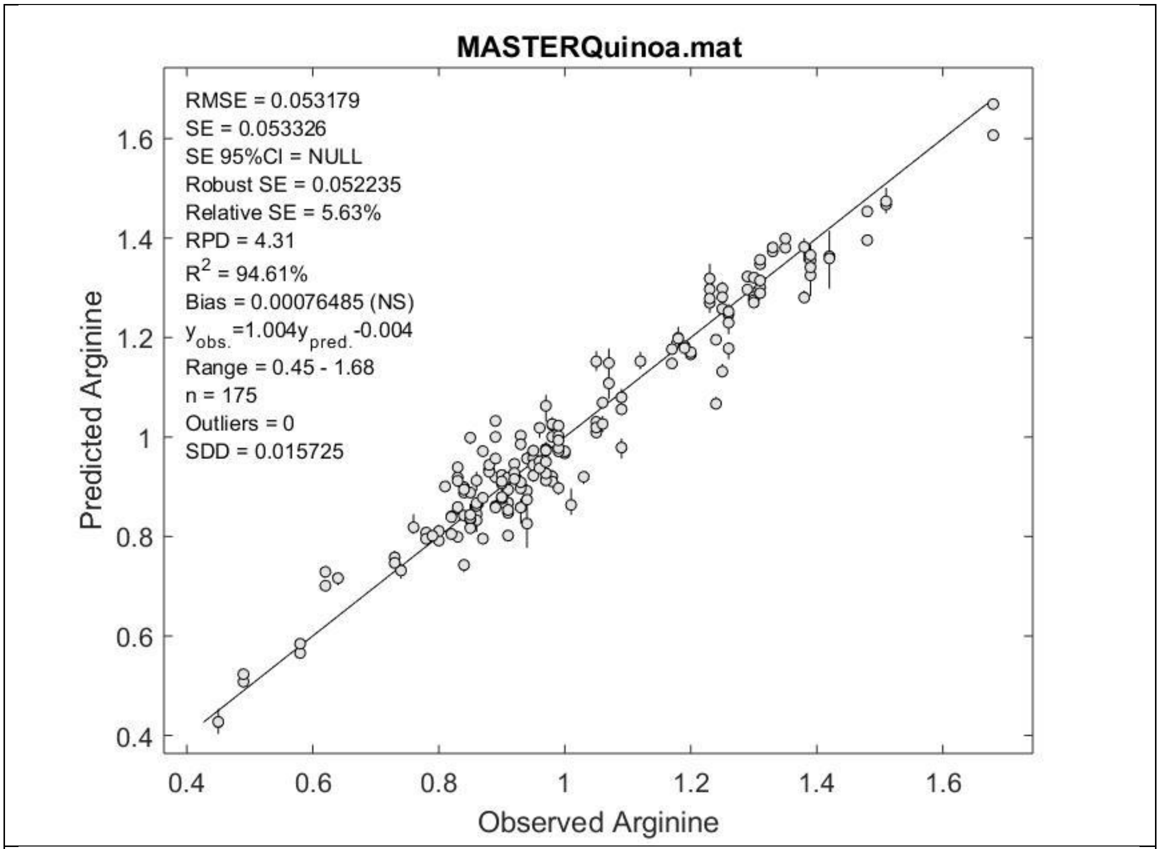
MASTERQuinoa.mat
RMSE = 0.053179
SE = 0.053326
SE 95%CI = NULL
Robust SE = 0.052235
Relative SE = 5.63%
RPD = 4.31
R2 = 94.61%
Bias = 0.00076485 (NS)
yobs.=1.004ypred.-0.004
Range = 0.45 - 1.68
n = 175
Outliers = 0
SDD = 0.015725
Predicted Arginine
Observed Arginine

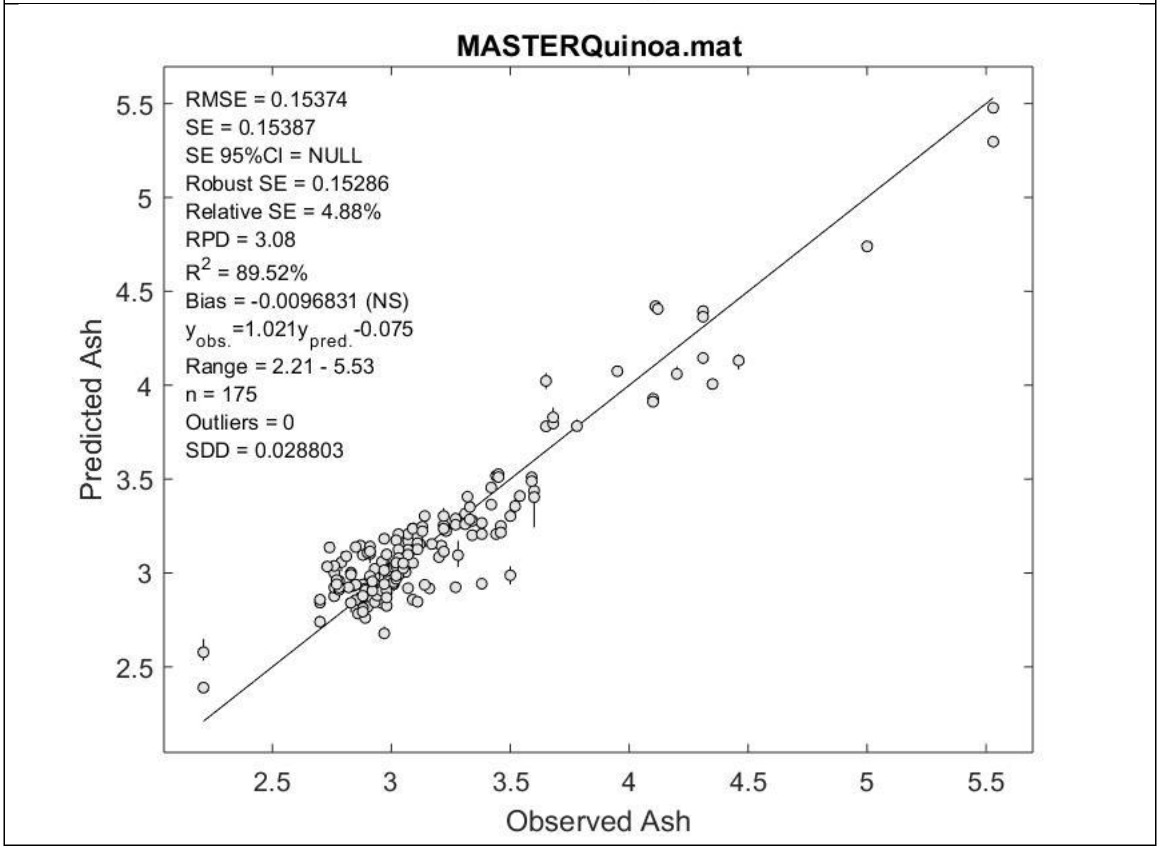
MASTERQuinoa.mat
RMSE = 0.15374
SE = 0.15387
SE 95%CI = NULL
Robust SE = 0.15286
Relative SE = 4.88%
RPD = 3.08
R2 = 89.52%
Bias = -0.0096831 (NS)
yobs.=1.021ypred.-0.075
Range = 2.21 - 5.53
n = 175
Outliers = 0
SDD = 0.028803
Predicted Ash
Observed Ash

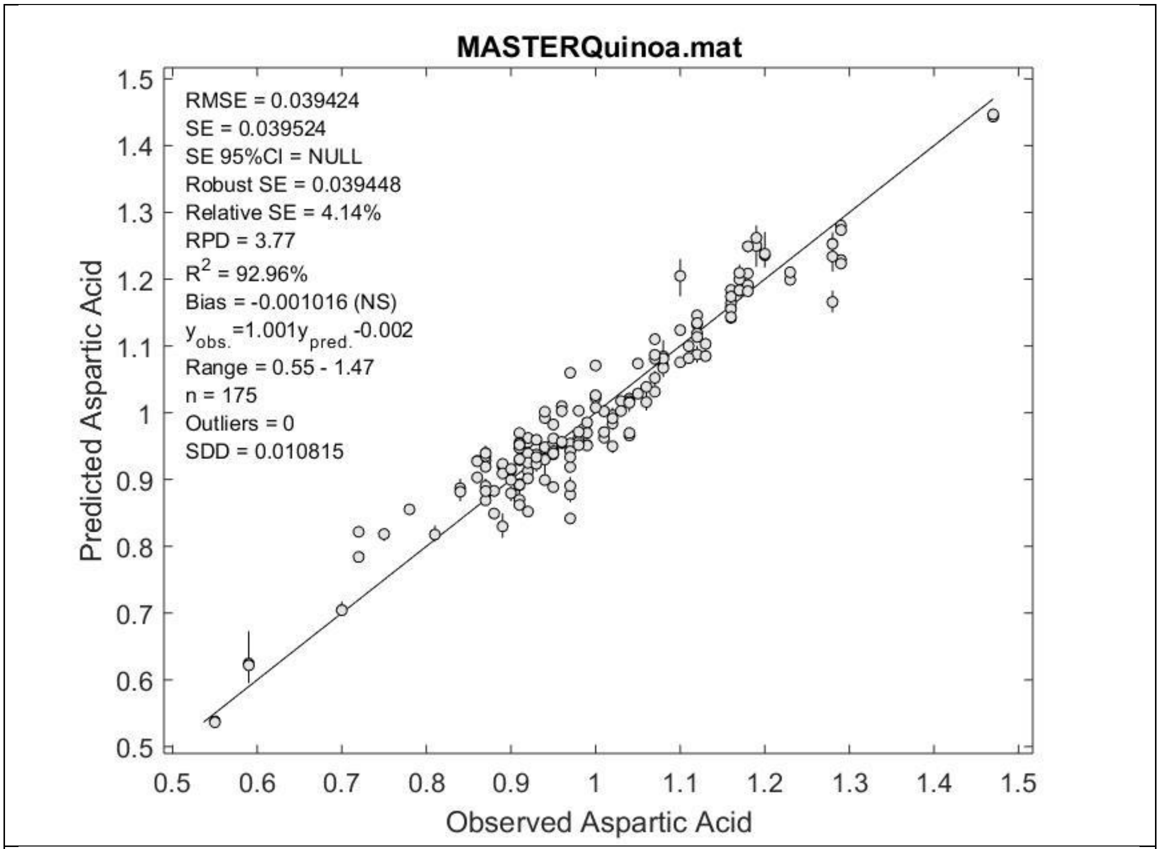
MASTERQuinoa.mat
RMSE = 0.039424
SE = 0.039524
SE 95%CI = NULL
Robust SE = 0.039448
Relative SE = 4.14%
RPD = 3.77
R^2 = 92.96%
Bias = -0.001016 (NS)
$y_{obs.}$=1.001$y_{pred.}$-0.002
Range = 0.55 - 1.47
n = 175
Outliers = 0
SDD = 0.010815
Predicted Aspartic Acid
Observed Aspartic Acid

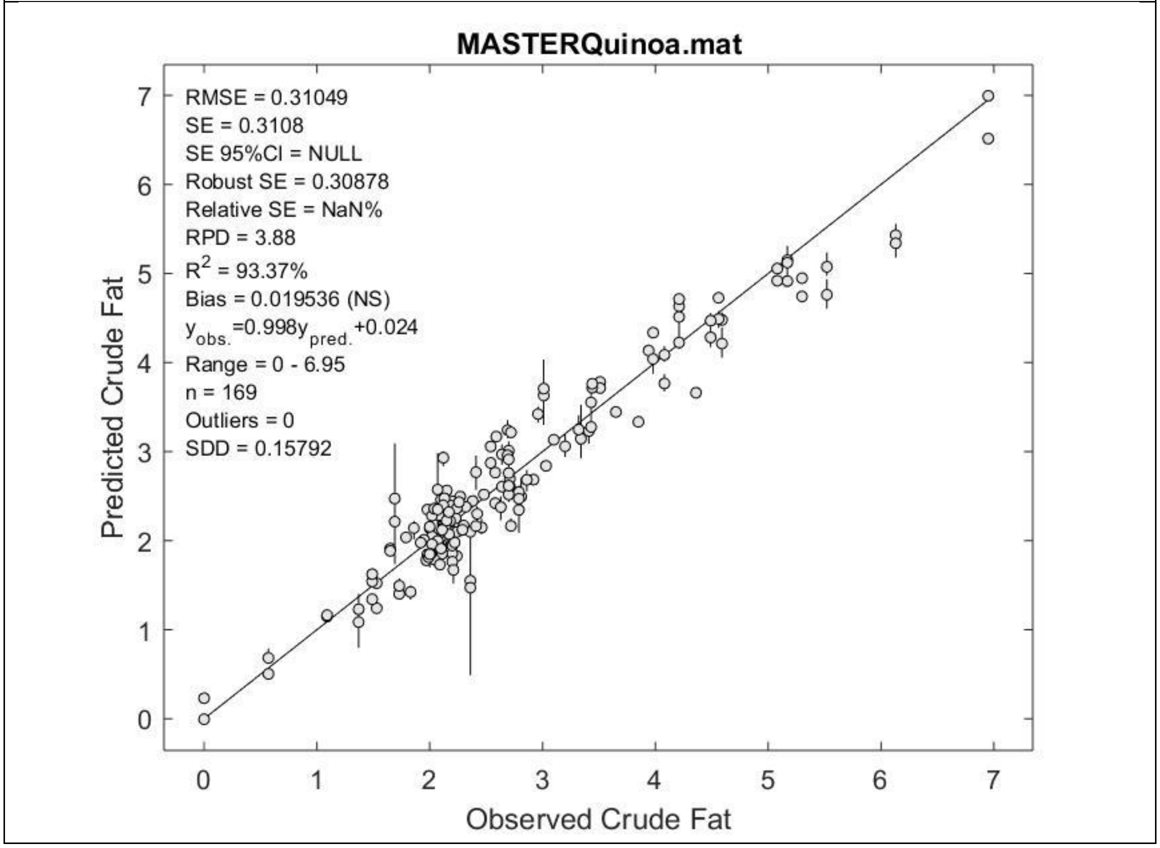
MASTERQuinoa.mat
RMSE = 0.31049
SE = 0.3108
SE 95%CI = NULL
Robust SE = 0.30878
Relative SE = NaN%
RPD = 3.88
R^2 = 93.37%
Bias = 0.019536 (NS)
$y_{obs.}$=0.998$y_{pred.}$+0.024
Range = 0 - 6.95
n = 169
Outliers = 0
SDD = 0.15792
Predicted Crude Fat
Observed Crude Fat

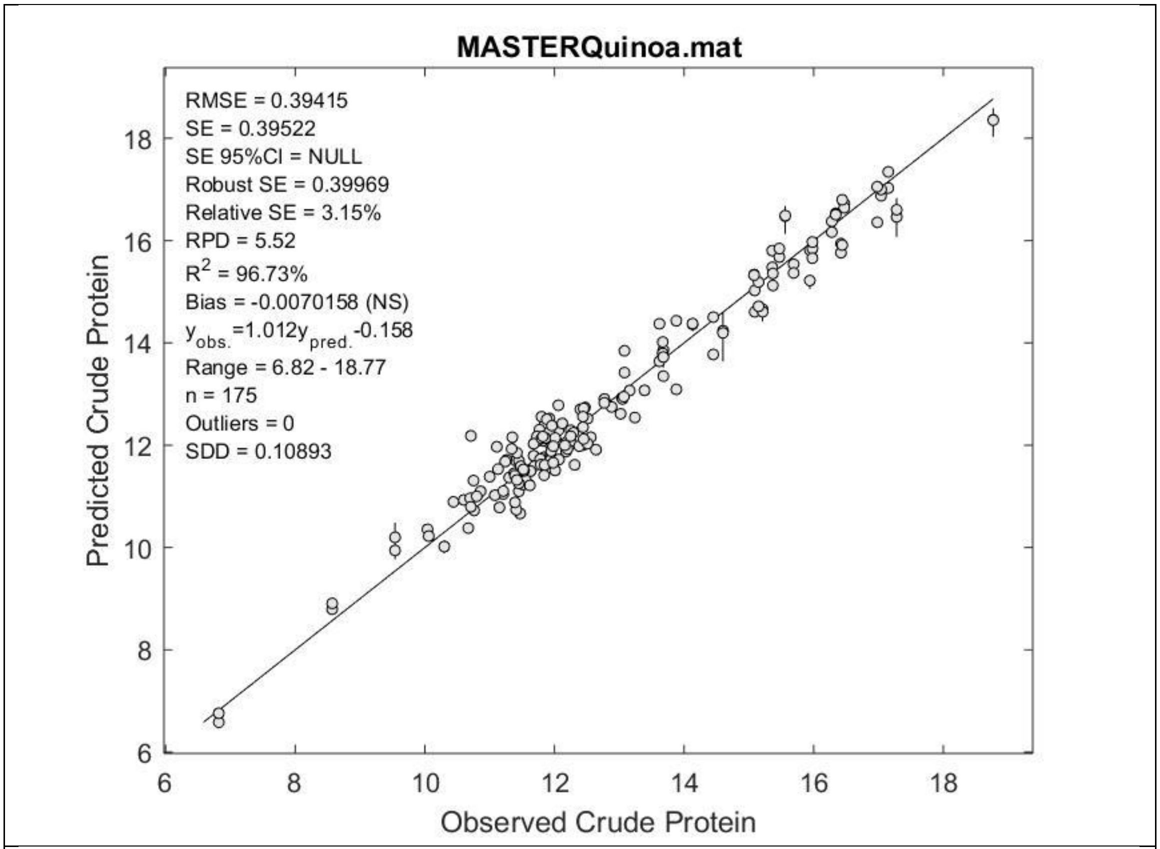
MASTERQuinoa.mat
RMSE = 0.39415
SE = 0.39522
SE 95%CI = NULL
Robust SE = 0.39969
Relative SE = 3.15%
RPD = 5.52
R2 = 96.73%
Bias = -0.0070158 (NS)
yobs.=1.012ypred.-0.158
Range = 6.82 - 18.77
n = 175
Outliers = 0
SDD = 0.10893
Predicted Crude Protein
Observed Crude Protein

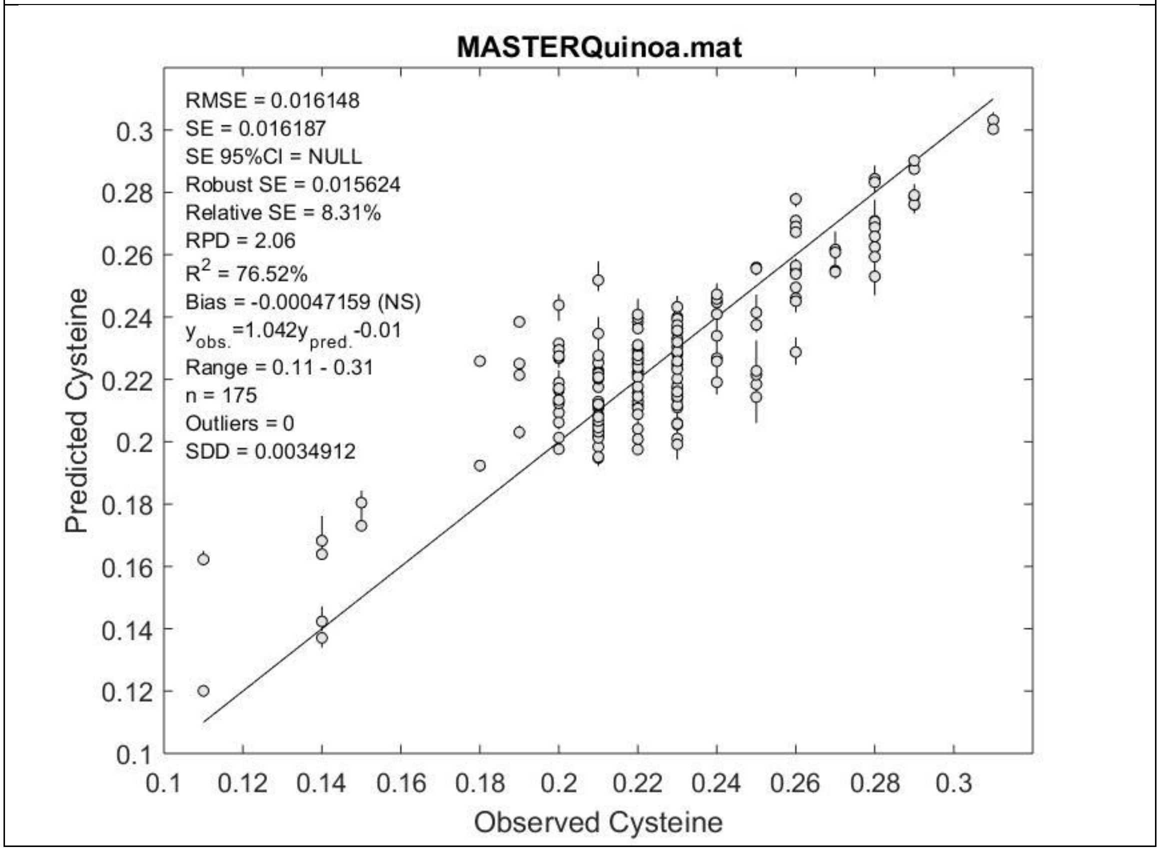
MASTERQuinoa.mat
RMSE = 0.016148
SE = 0.016187
SE 95%CI = NULL
Robust SE = 0.015624
Relative SE = 8.31%
RPD = 2.06
R2 = 76.52%
Bias = -0.00047159 (NS)
yobs.=1.042ypred.-0.01
Range = 0.11 - 0.31
n = 175
Outliers = 0
SDD = 0.0034912
Predicted Cysteine
Observed Cysteine

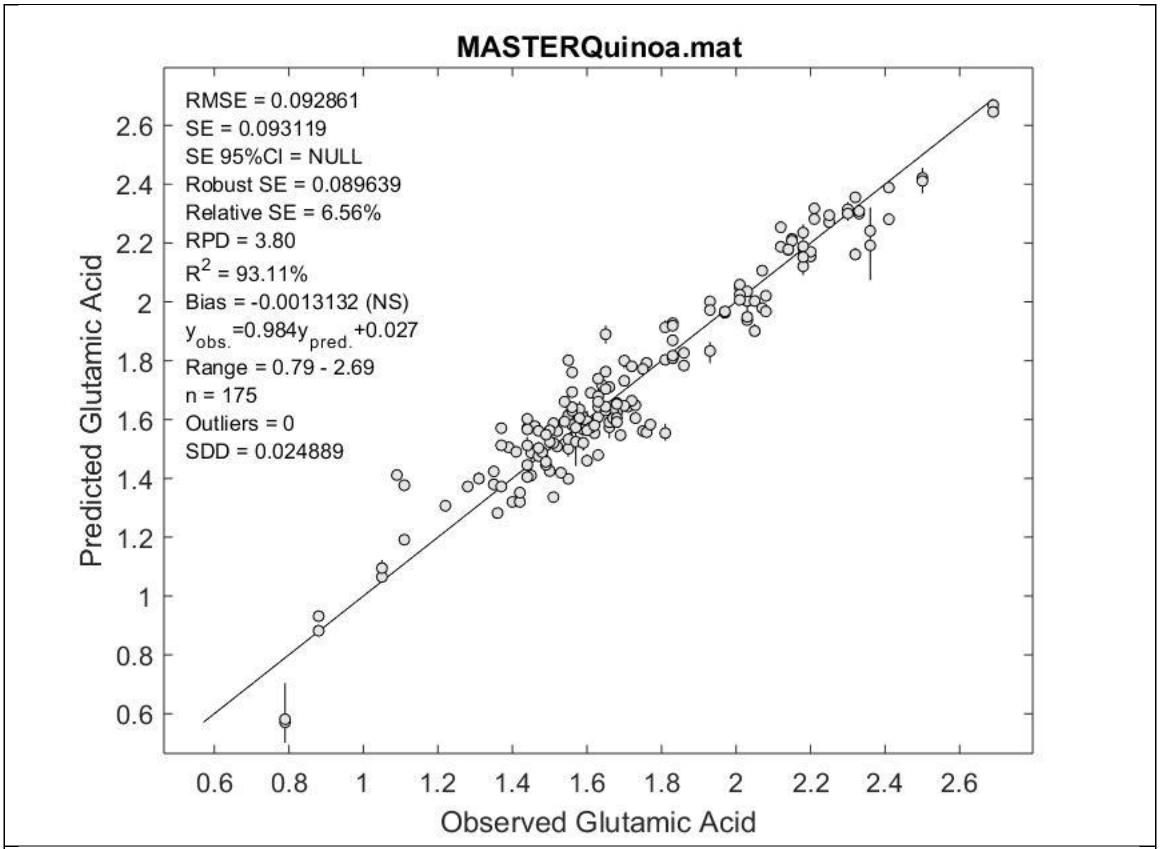

MASTERQuinoa.mat
RMSE = 0.092861
SE = 0.093119
SE 95%CI = NULL
Robust SE = 0.089639
Relative SE = 6.56%
RPD = 3.80
R^2 = 93.11%
Bias = -0.0013132 (NS)
$y_{obs.}$=0.984$y_{pred.}$+0.027
Range = 0.79 - 2.69
n = 175
Outliers = 0
SDD = 0.024889
Predicted Glutamic Acid
Observed Glutamic Acid

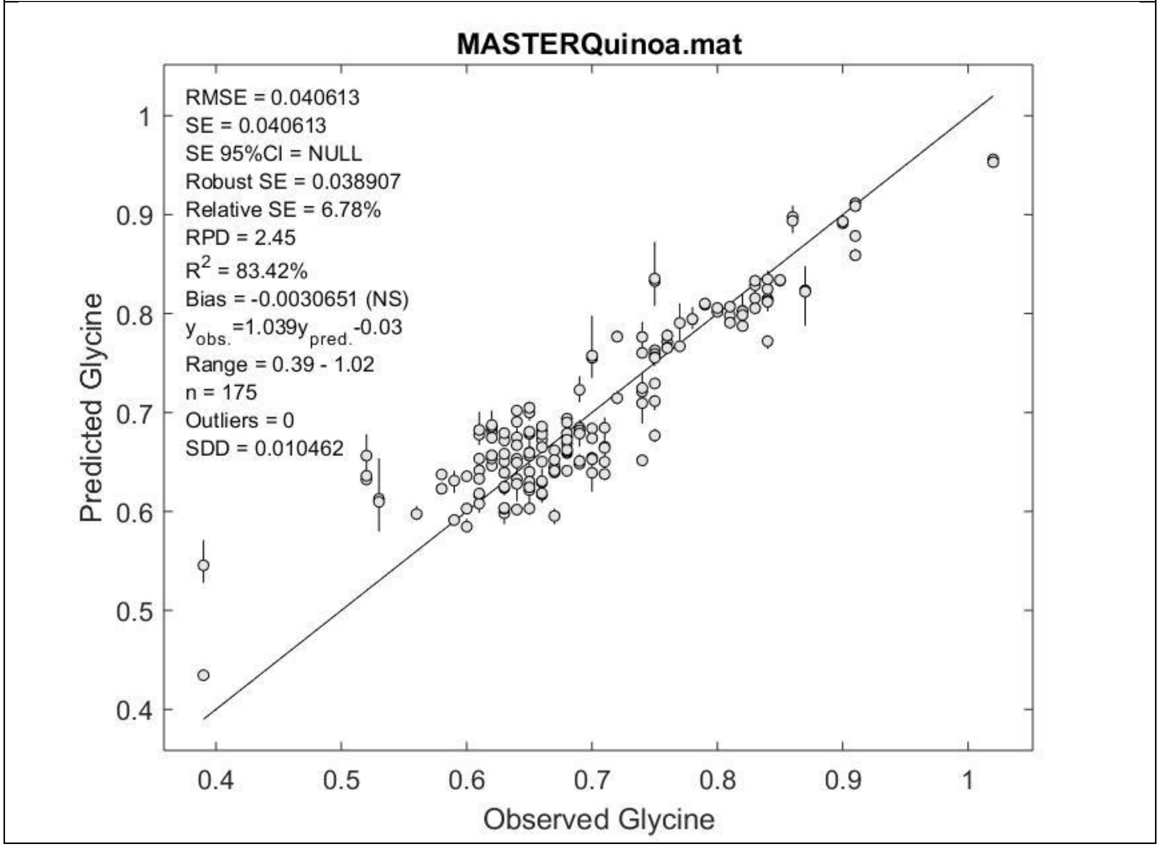

MASTERQuinoa.mat
RMSE = 0.040613
SE = 0.040613
SE 95%CI = NULL
Robust SE = 0.038907
Relative SE = 6.78%
RPD = 2.45
R^2 = 83.42%
Bias = -0.0030651 (NS)
$y_{obs.}$=1.039$y_{pred.}$-0.03
Range = 0.39 - 1.02
n = 175
Outliers = 0
SDD = 0.010462
Predicted Glycine
Observed Glycine

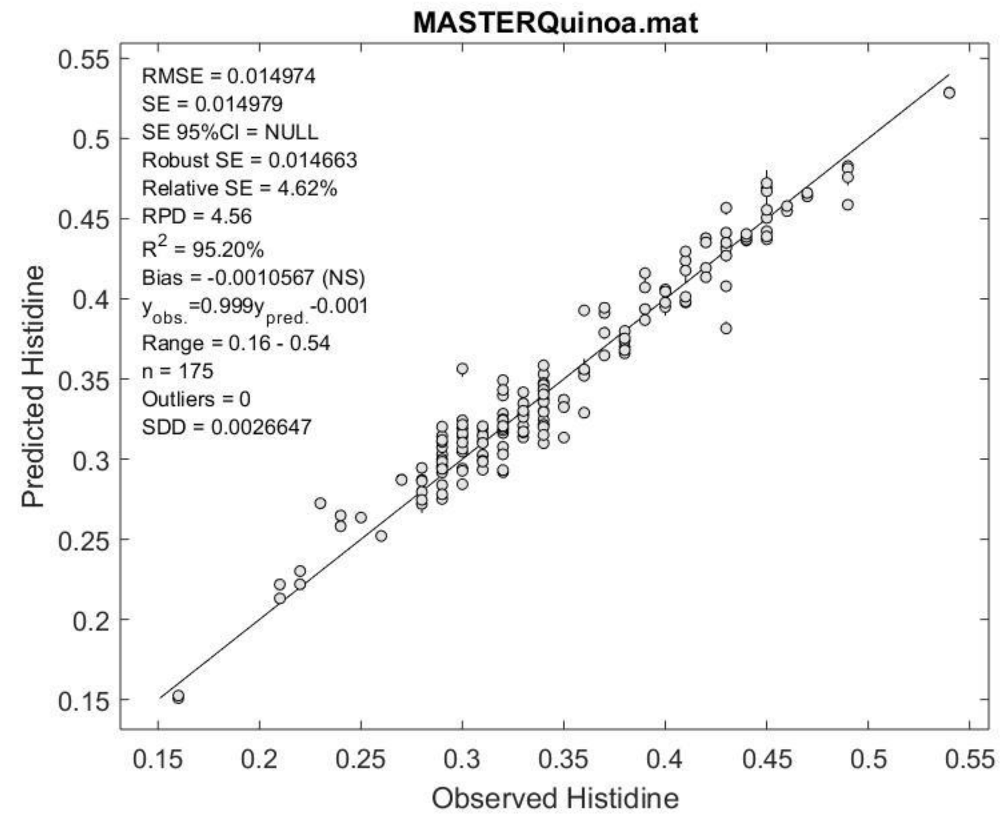
MASTERQuinoa.mat
RMSE = 0.014974
SE = 0.014979
SE 95%CI = NULL
Robust SE = 0.014663
Relative SE = 4.62%
RPD = 4.56
R2 = 95.20%
Bias = -0.0010567 (NS)
yobs.=0.999ypred.-0.001
Range = 0.16 - 0.54
n = 175
Outliers = 0
SDD = 0.0026647
Predicted Histidine
Observed Histidine
0.15
0.2
0.25
0.3
0.35
0.4
0.45
0.5
0.55

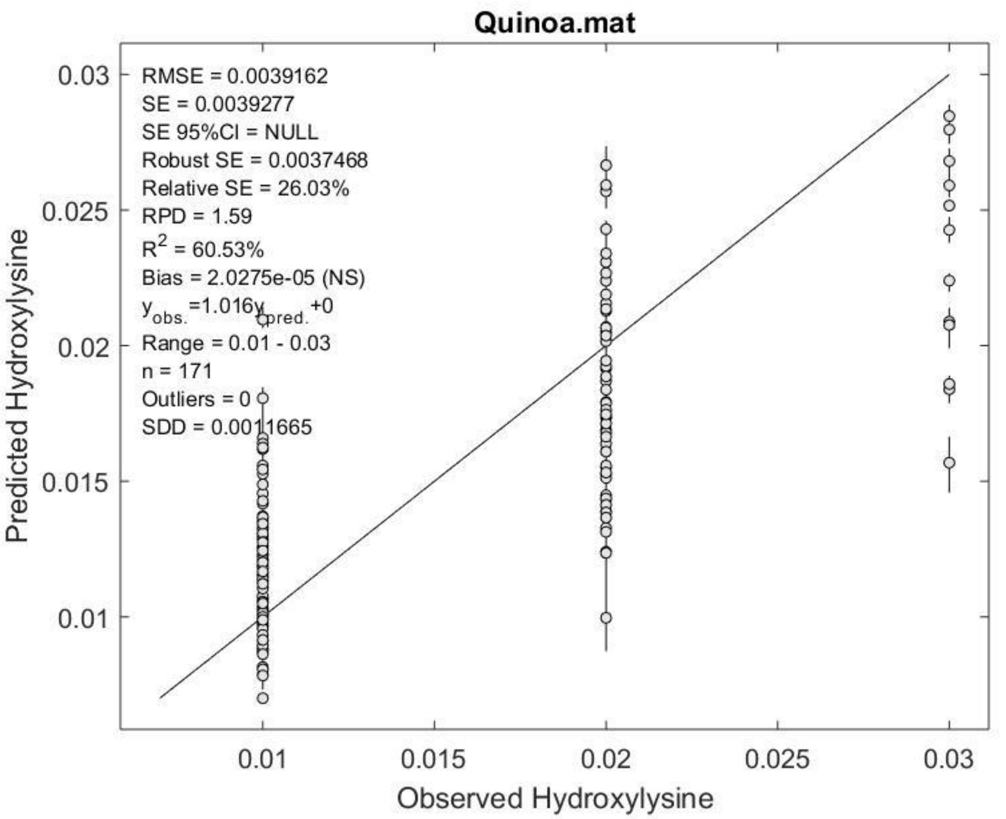
Quinoa.mat
RMSE = 0.0039162
SE = 0.0039277
SE 95%CI = NULL
Robust SE = 0.0037468
Relative SE = 26.03%
RPD = 1.59
R2 = 60.53%
Bias = 2.0275e-05 (NS)
yobs.=1.016ypred.+0
Range = 0.01 - 0.03
n = 171
Outliers = 0
SDD = 0.0011665
Predicted Hydroxylysine
Observed Hydroxylysine
0.01
0.015
0.02
0.025
0.03

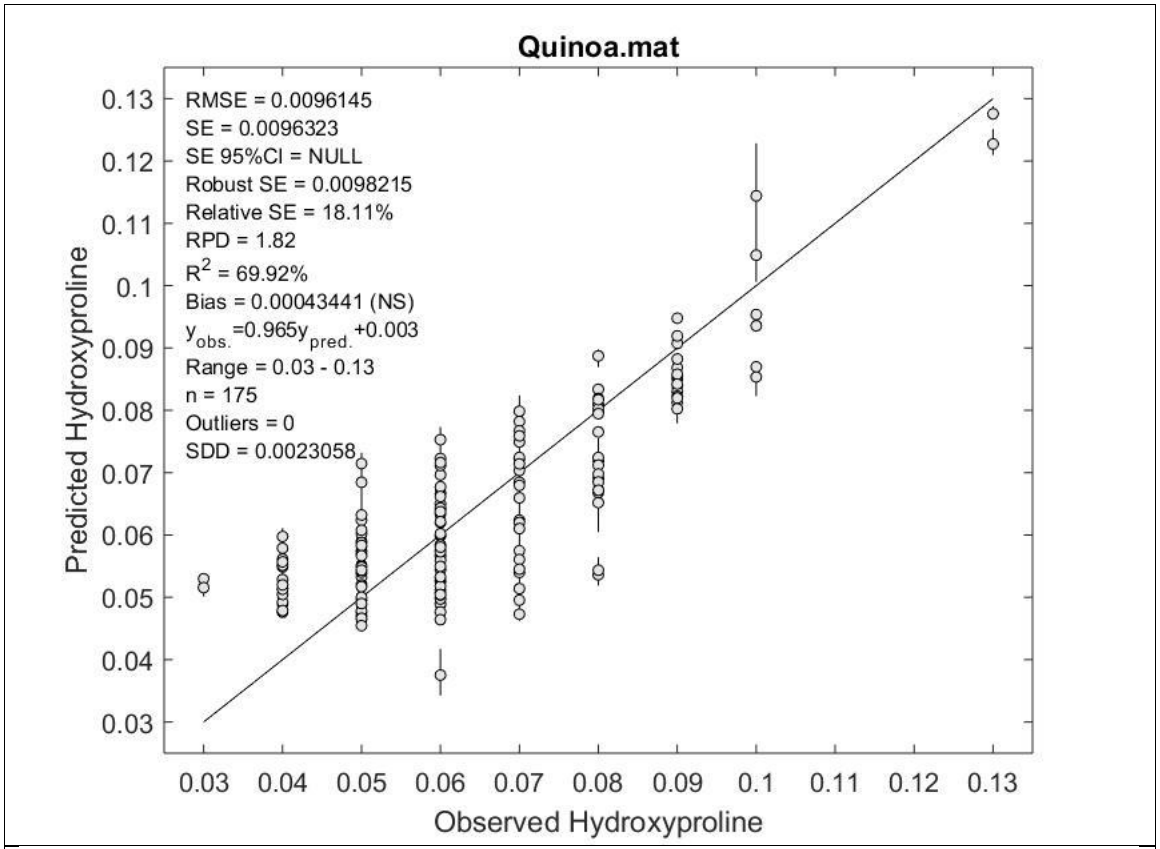
Quinoa.mat
RMSE = 0.0096145
SE = 0.0096323
SE 95%CI = NULL
Robust SE = 0.0098215
Relative SE = 18.11%
RPD = 1.82
R^2 = 69.92%
Bias = 0.00043441 (NS)
$y_{obs.}$=0.965$y_{pred.}$+0.003
Range = 0.03 - 0.13
n = 175
Outliers = 0
SDD = 0.0023058
Predicted Hydroxyproline
Observed Hydroxyproline

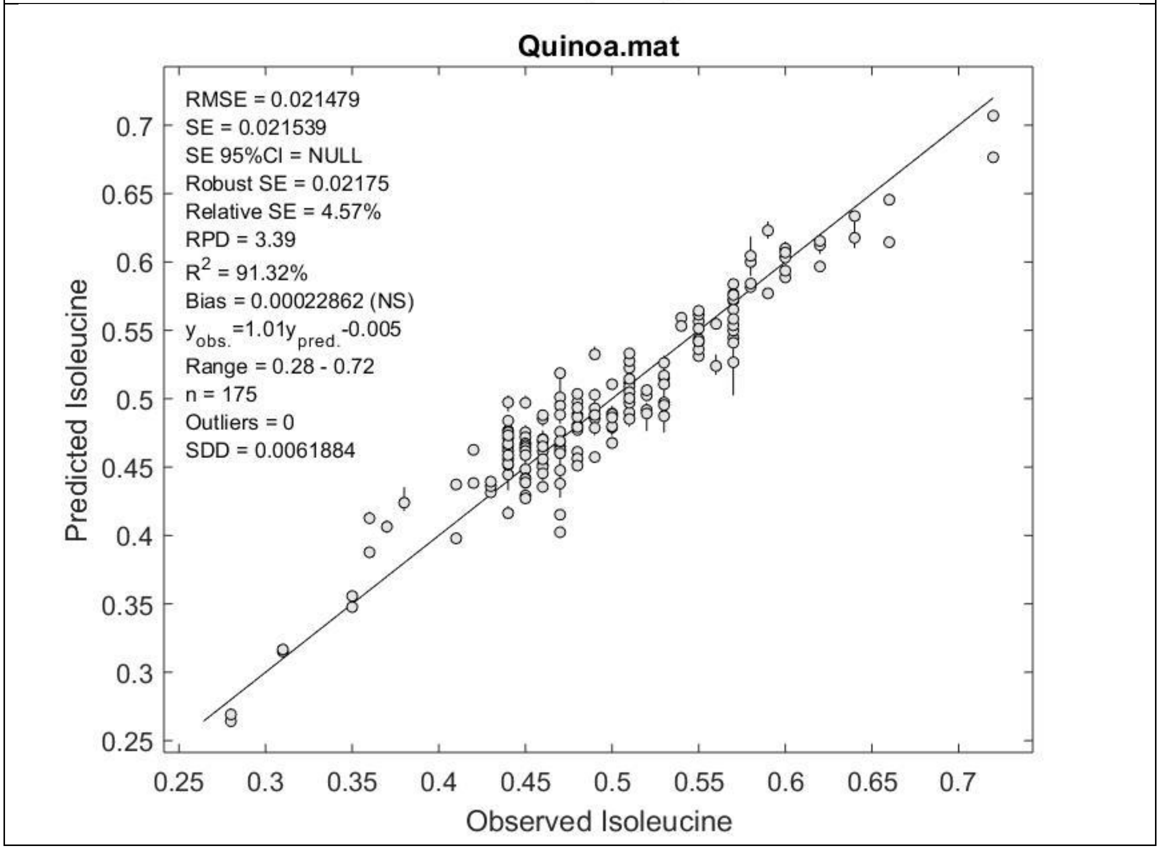
Quinoa.mat
RMSE = 0.021479
SE = 0.021539
SE 95%CI = NULL
Robust SE = 0.02175
Relative SE = 4.57%
RPD = 3.39
R^2 = 91.32%
Bias = 0.00022862 (NS)
$y_{obs.}$=1.01$y_{pred.}$-0.005
Range = 0.28 - 0.72
n = 175
Outliers = 0
SDD = 0.0061884
Predicted Isoleucine
Observed Isoleucine

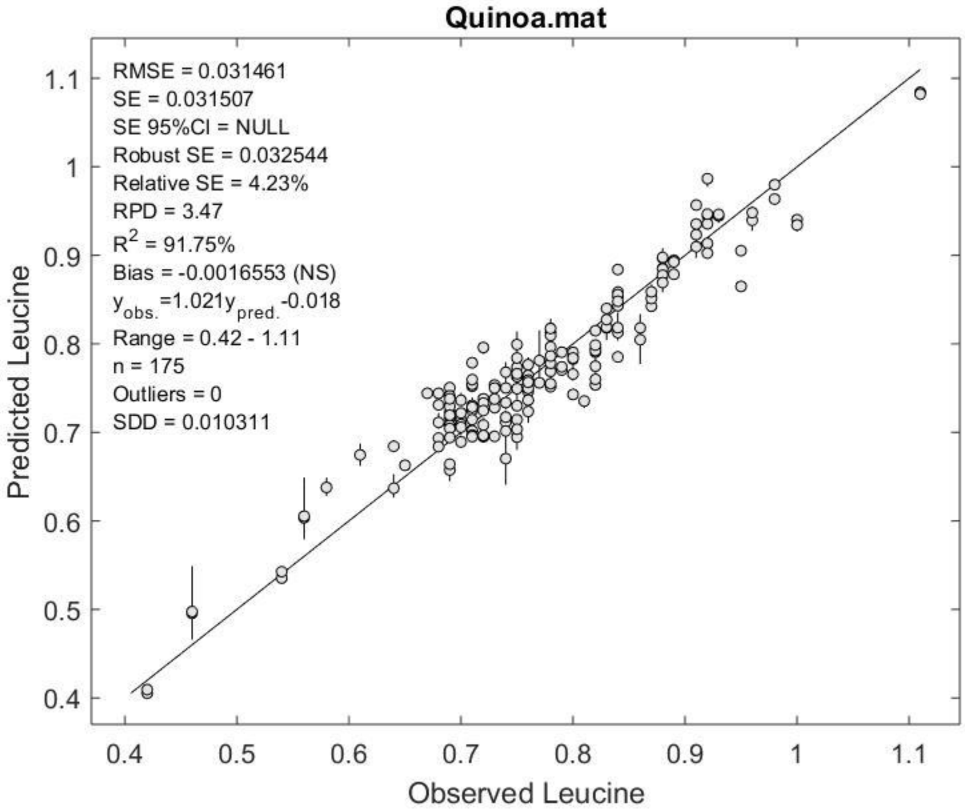
Quinoa.mat
RMSE = 0.031461
SE = 0.031507
SE 95%CI = NULL
Robust SE = 0.032544
Relative SE = 4.23%
RPD = 3.47
R^2 = 91.75%
Bias = -0.0016553 (NS)
$y_{obs.}$=1.021$y_{pred.}$-0.018
Range = 0.42 - 1.11
n = 175
Outliers = 0
SDD = 0.010311
Predicted Leucine
Observed Leucine

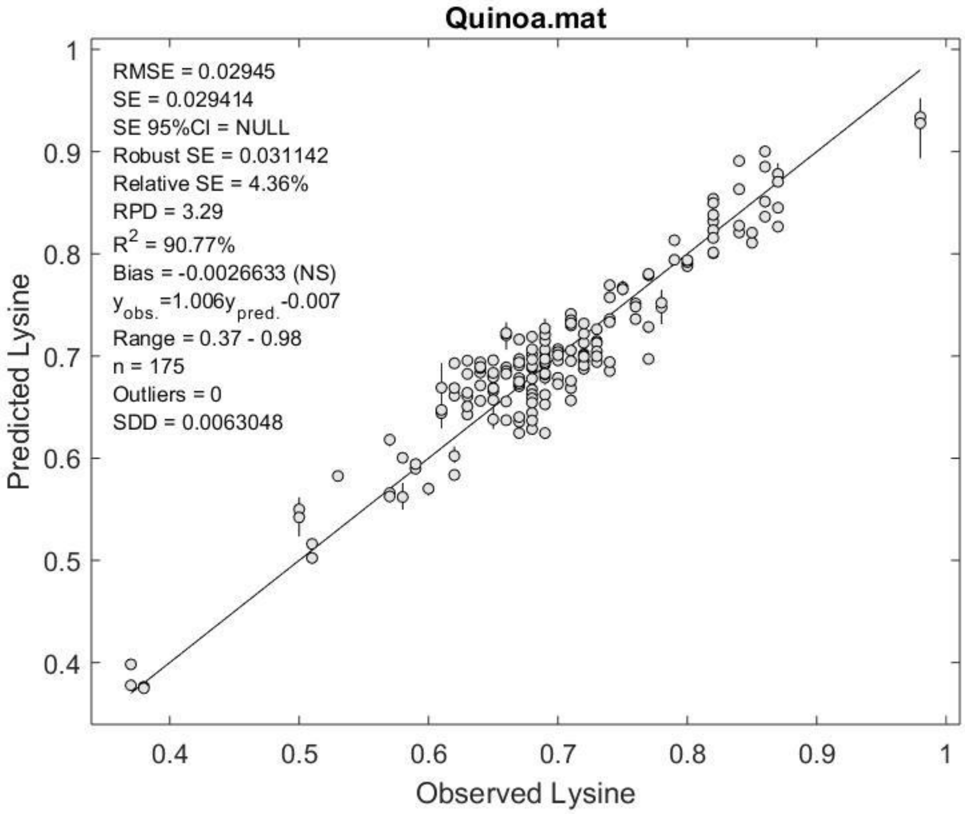
Quinoa.mat
RMSE = 0.02945
SE = 0.029414
SE 95%CI = NULL
Robust SE = 0.031142
Relative SE = 4.36%
RPD = 3.29
R^2 = 90.77%
Bias = -0.0026633 (NS)
$y_{obs.}$=1.006$y_{pred.}$-0.007
Range = 0.37 - 0.98
n = 175
Outliers = 0
SDD = 0.0063048
Predicted Lysine
Observed Lysine

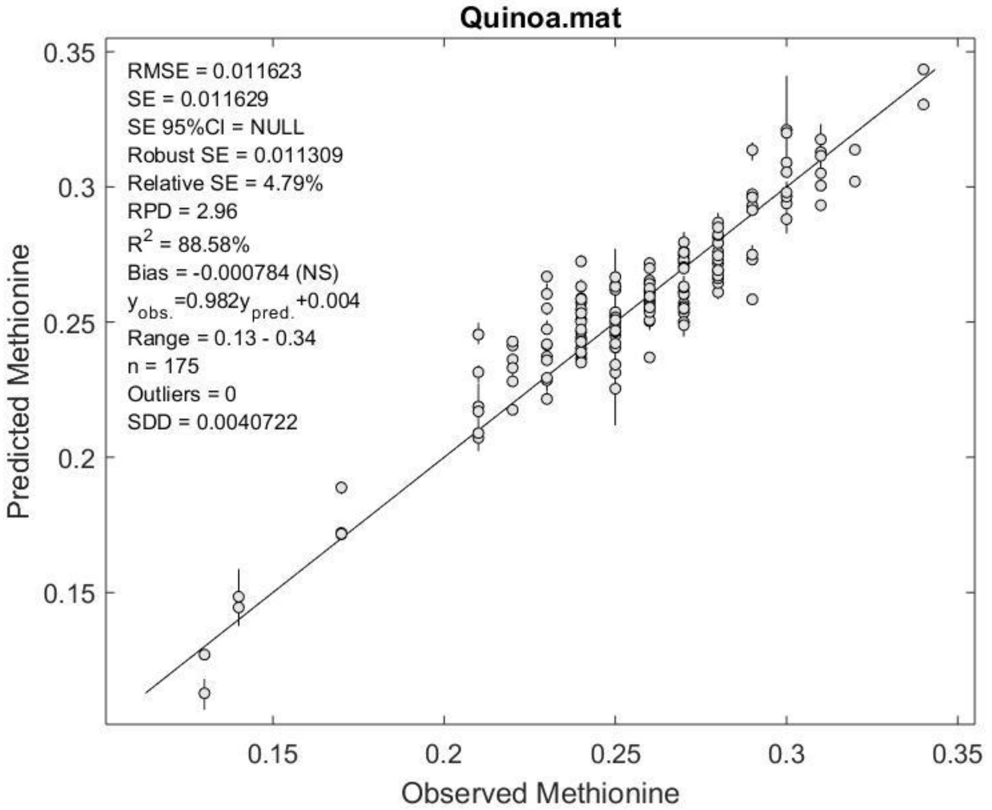
Quinoa.mat
RMSE = 0.011623
SE = 0.011629
SE 95%CI = NULL
Robust SE = 0.011309
Relative SE = 4.79%
RPD = 2.96
R^2 = 88.58%
Bias = -0.000784 (NS)
$y_{obs.}$=0.982$y_{pred.}$+0.004
Range = 0.13 - 0.34
n = 175
Outliers = 0
SDD = 0.0040722
Predicted Methionine
Observed Methionine
0.35
0.3
0.25
0.2
0.15
0.15
0.2
0.25
0.3
0.35

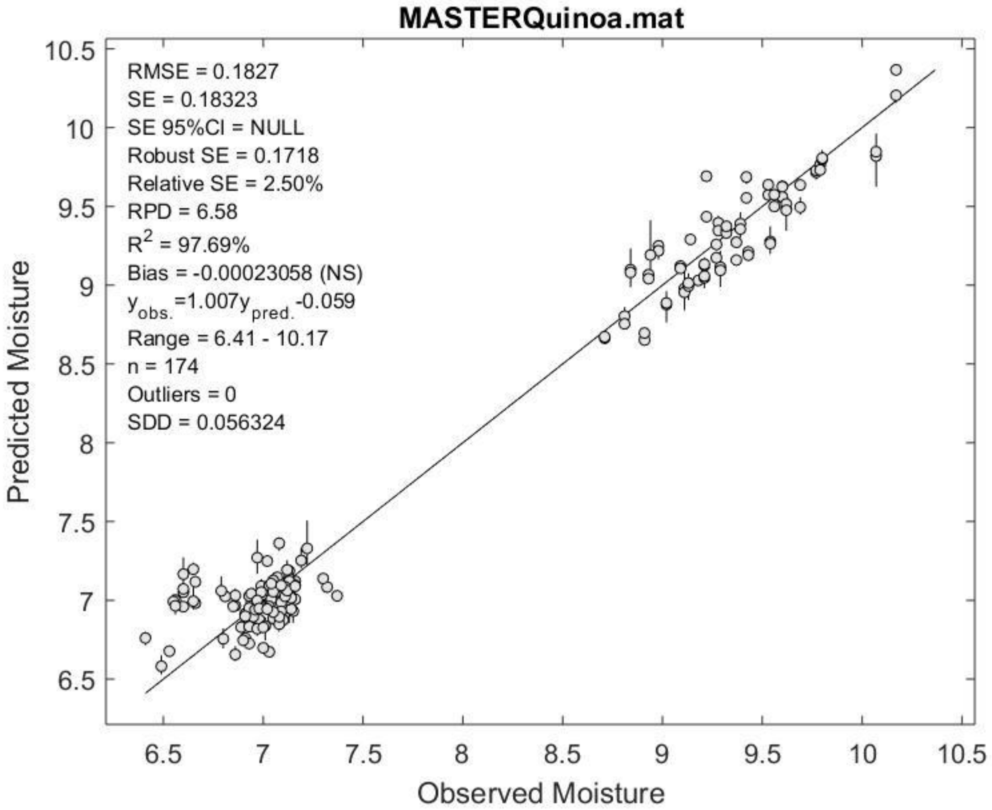
MASTERQuinoa.mat
RMSE = 0.1827
SE = 0.18323
SE 95%CI = NULL
Robust SE = 0.1718
Relative SE = 2.50%
RPD = 6.58
R^2 = 97.69%
Bias = -0.00023058 (NS)
$y_{obs.}$=1.007$y_{pred.}$-0.059
Range = 6.41 - 10.17
n = 174
Outliers = 0
SDD = 0.056324
Predicted Moisture
Observed Moisture
10.5
10
9.5
9
8.5
8
7.5
7
6.5
6.5
7
7.5
8
8.5
9
9.5
10
10.5

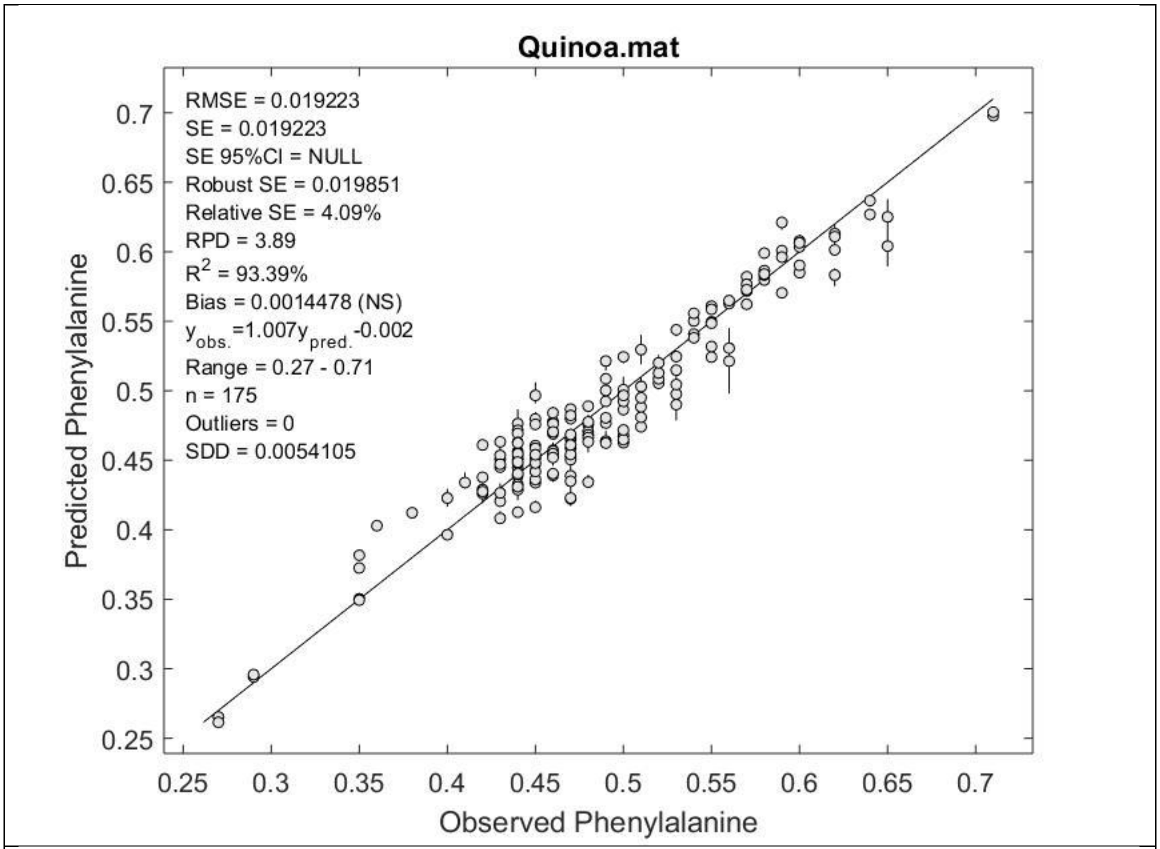
Quinoa.mat
RMSE = 0.019223
SE = 0.019223
SE 95%CI = NULL
Robust SE = 0.019851
Relative SE = 4.09%
RPD = 3.89
R2 = 93.39%
Bias = 0.0014478 (NS)
yobs.=1.007ypred.-0.002
Range = 0.27 - 0.71
n = 175
Outliers = 0
SDD = 0.0054105
Predicted Phenylalanine
Observed Phenylalanine
0.25 0.3 0.35 0.4 0.45 0.5 0.55 0.6 0.65 0.7

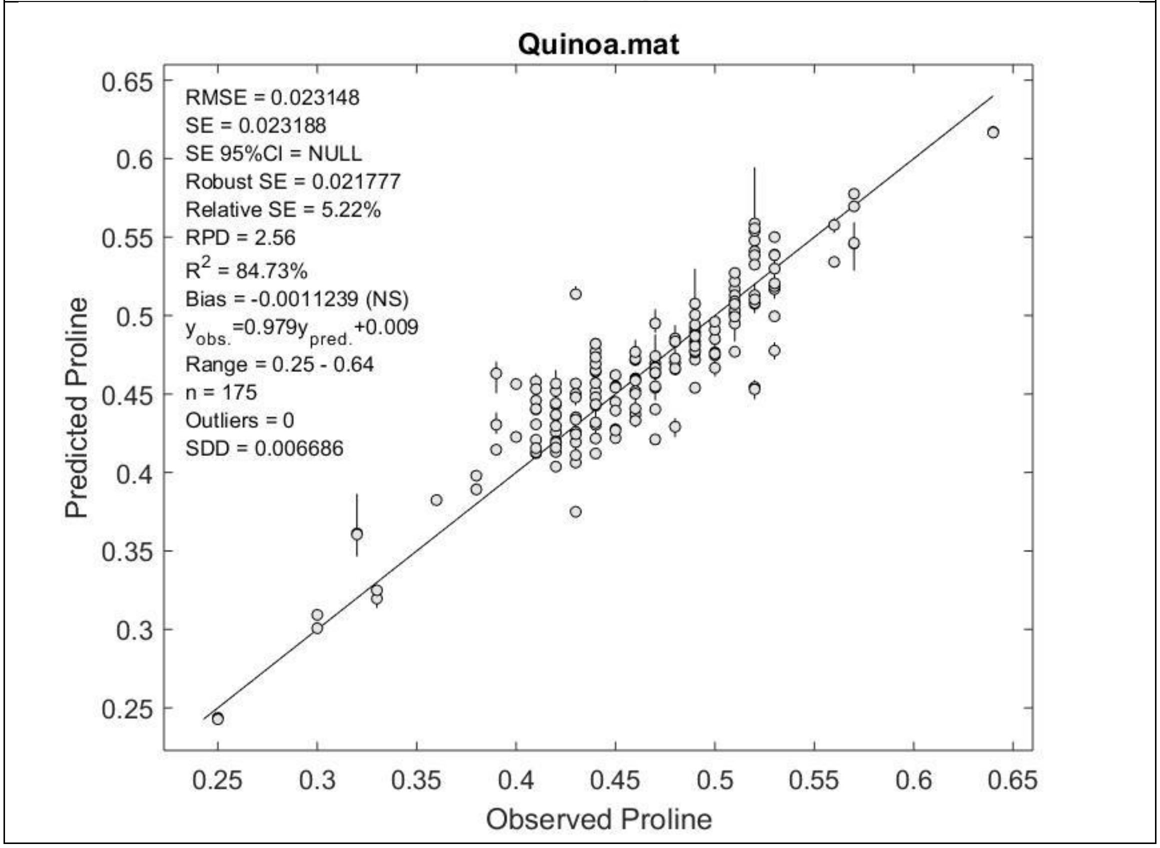
Quinoa.mat
RMSE = 0.023148
SE = 0.023188
SE 95%CI = NULL
Robust SE = 0.021777
Relative SE = 5.22%
RPD = 2.56
R2 = 84.73%
Bias = -0.0011239 (NS)
yobs.=0.979ypred.+0.009
Range = 0.25 - 0.64
n = 175
Outliers = 0
SDD = 0.006686
Predicted Proline
Observed Proline
0.25 0.3 0.35 0.4 0.45 0.5 0.55 0.6 0.65

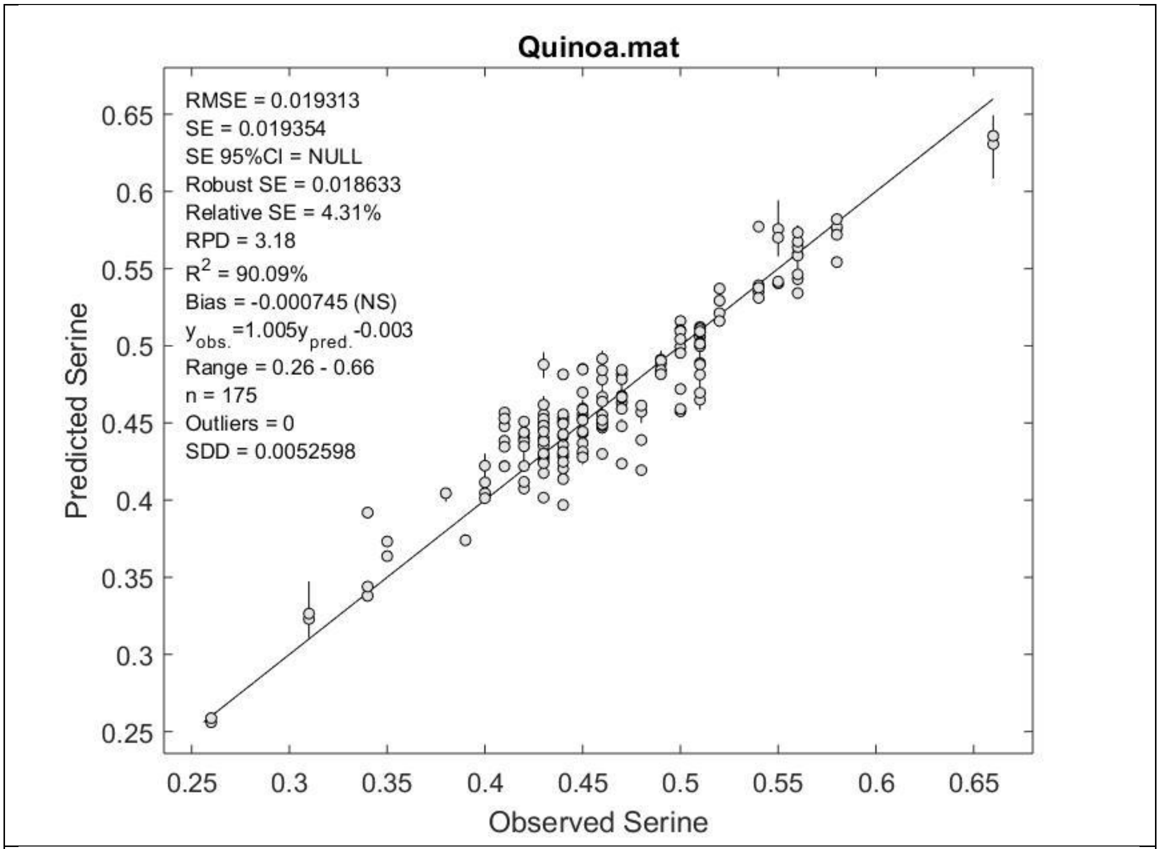
Quinoa.mat
RMSE = 0.019313
SE = 0.019354
SE 95%CI = NULL
Robust SE = 0.018633
Relative SE = 4.31%
RPD = 3.18
R2 = 90.09%
Bias = -0.000745 (NS)
yobs.=1.005ypred.-0.003
Range = 0.26 - 0.66
n = 175
Outliers = 0
SDD = 0.0052598
Predicted Serine
Observed Serine

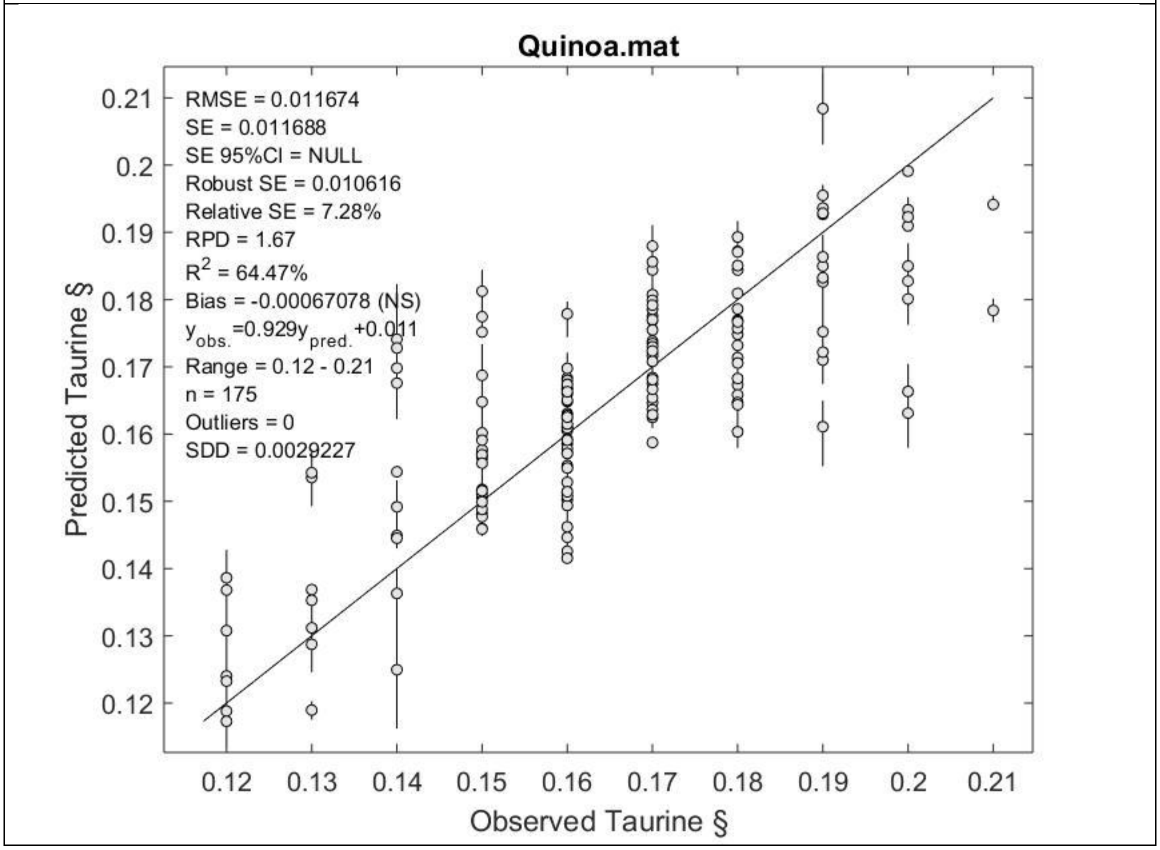
Quinoa.mat
RMSE = 0.011674
SE = 0.011688
SE 95%CI = NULL
Robust SE = 0.010616
Relative SE = 7.28%
RPD = 1.67
R2 = 64.47%
Bias = -0.00067078 (NS)
yobs.=0.929ypred.+0.011
Range = 0.12 - 0.21
n = 175
Outliers = 0
SDD = 0.0029227
Predicted Taurine §
Observed Taurine §

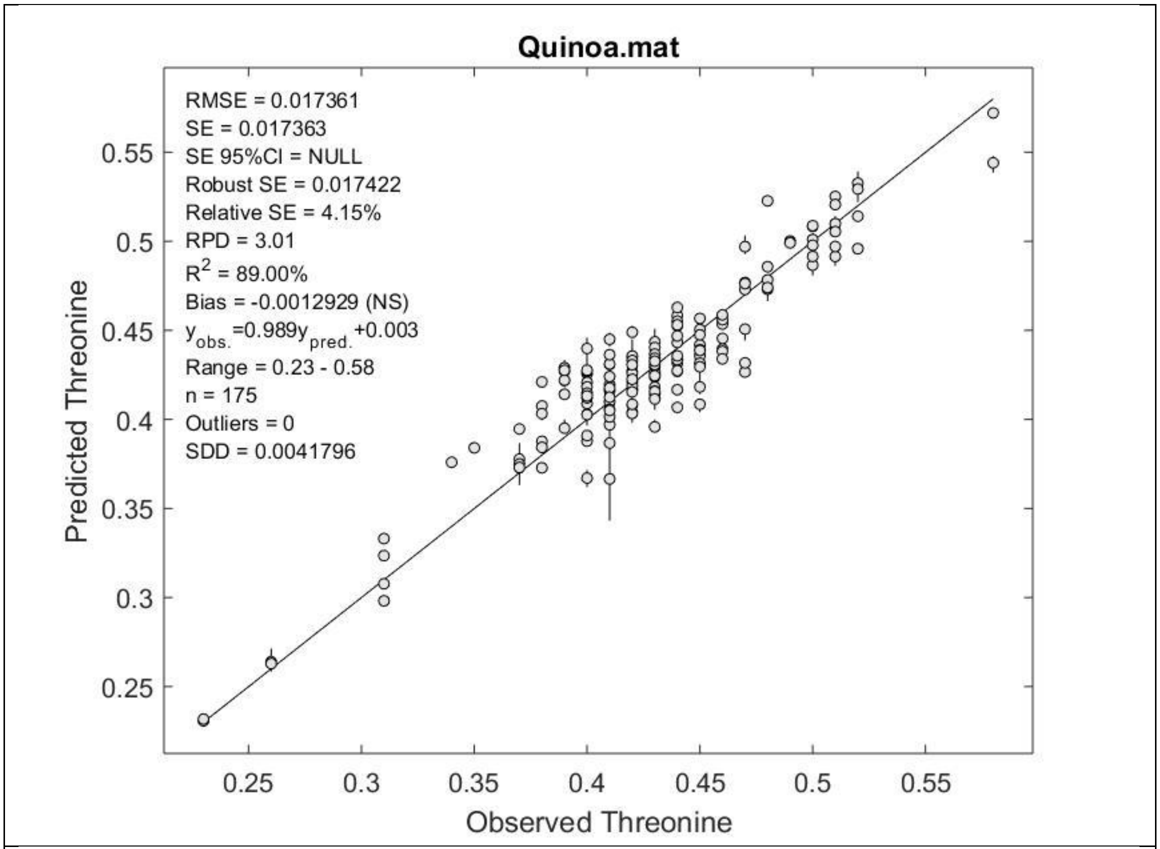
Quinoa.mat
RMSE = 0.017361
SE = 0.017363
SE 95%CI = NULL
Robust SE = 0.017422
Relative SE = 4.15%
RPD = 3.01
R2 = 89.00%
Bias = -0.0012929 (NS)
yobs.=0.989ypred.+0.003
Range = 0.23 - 0.58
n = 175
Outliers = 0
SDD = 0.0041796
Predicted Threonine
Observed Threonine

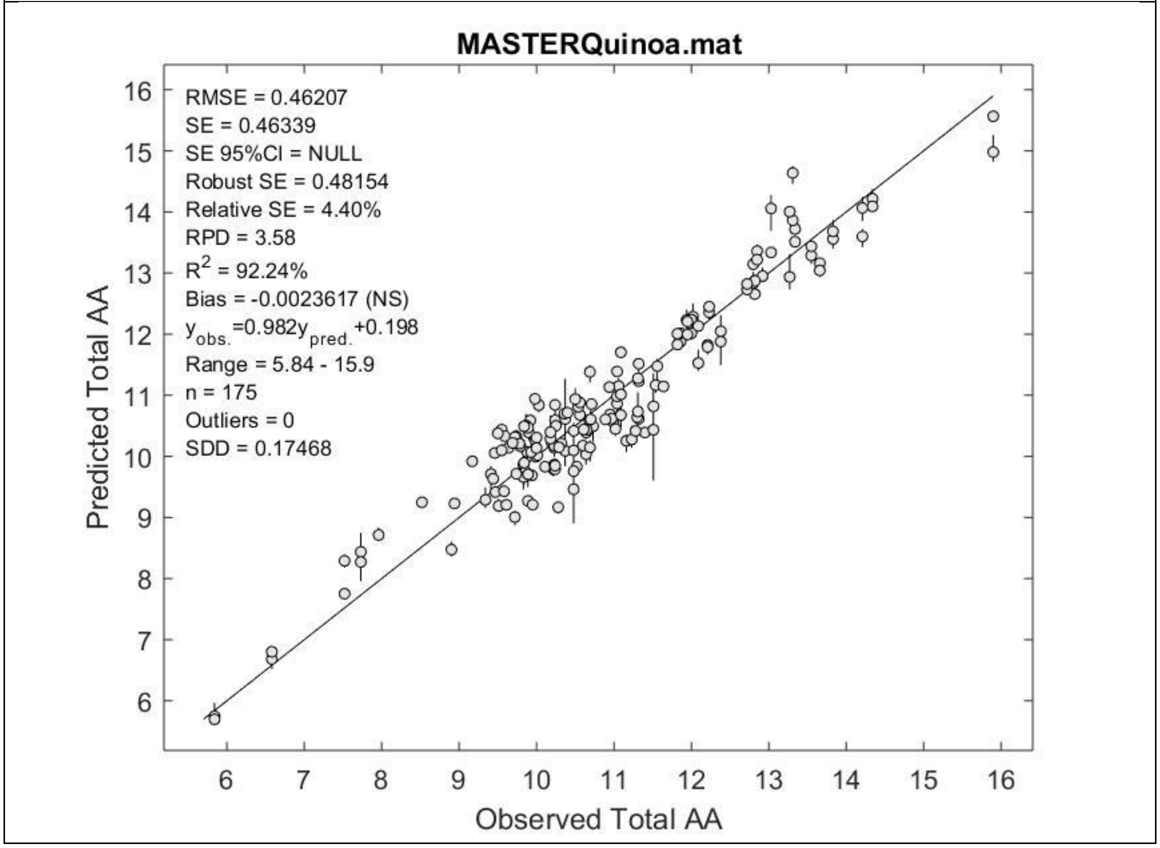
MASTERQuinoa.mat
RMSE = 0.46207
SE = 0.46339
SE 95%CI = NULL
Robust SE = 0.48154
Relative SE = 4.40%
RPD = 3.58
R2 = 92.24%
Bias = -0.0023617 (NS)
yobs.=0.982ypred.+0.198
Range = 5.84 - 15.9
n = 175
Outliers = 0
SDD = 0.17468
Predicted Total AA
Observed Total AA

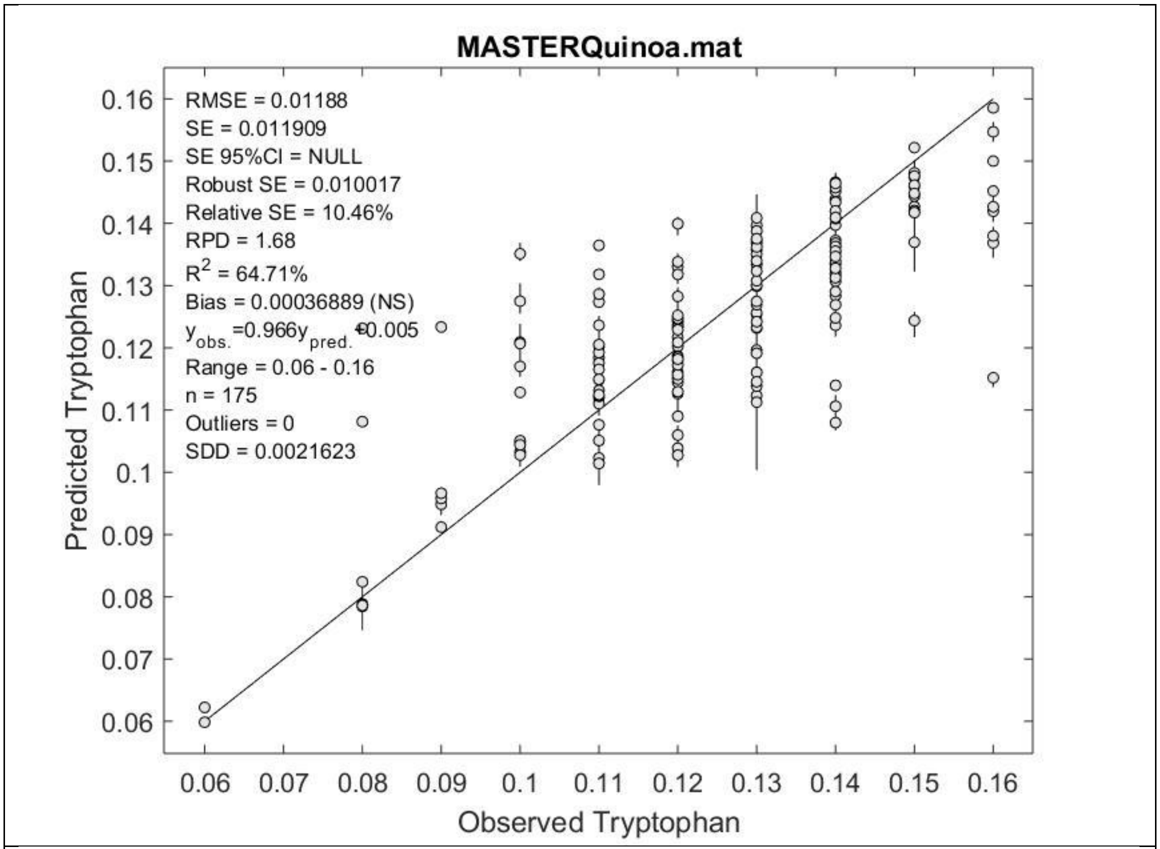
MASTERQuinoa.mat
RMSE = 0.01188
SE = 0.011909
SE 95%CI = NULL
Robust SE = 0.010017
Relative SE = 10.46%
RPD = 1.68
R2 = 64.71%
Bias = 0.00036889 (NS)
yobs.=0.966ypred.+0.005
Range = 0.06 - 0.16
n = 175
Outliers = 0
SDD = 0.0021623
Predicted Tryptophan
Observed Tryptophan
0.06
0.07
0.08
0.09
0.1
0.11
0.12
0.13
0.14
0.15
0.16

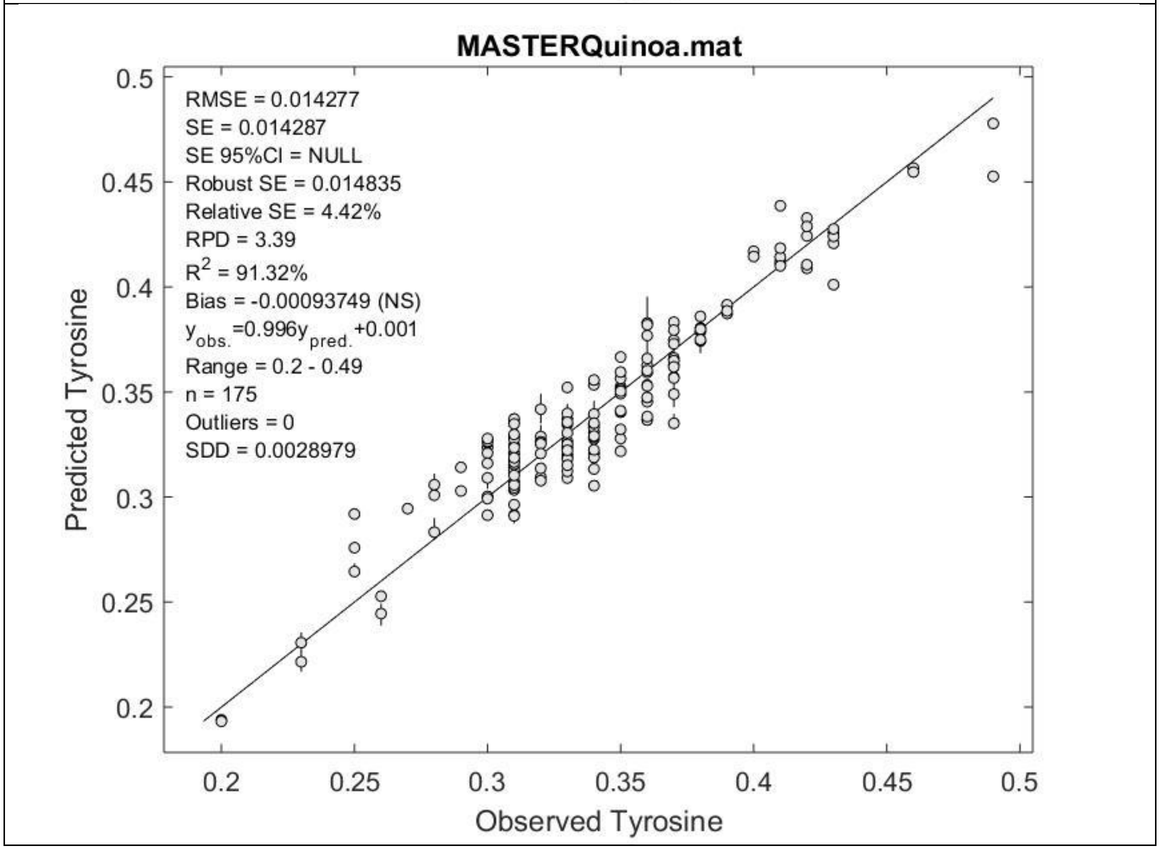
MASTERQuinoa.mat
RMSE = 0.014277
SE = 0.014287
SE 95%CI = NULL
Robust SE = 0.014835
Relative SE = 4.42%
RPD = 3.39
R2 = 91.32%
Bias = -0.00093749 (NS)
yobs.=0.996ypred.+0.001
Range = 0.2 - 0.49
n = 175
Outliers = 0
SDD = 0.0028979
Predicted Tyrosine
Observed Tyrosine
0.2
0.25
0.3
0.35
0.4
0.45
0.5

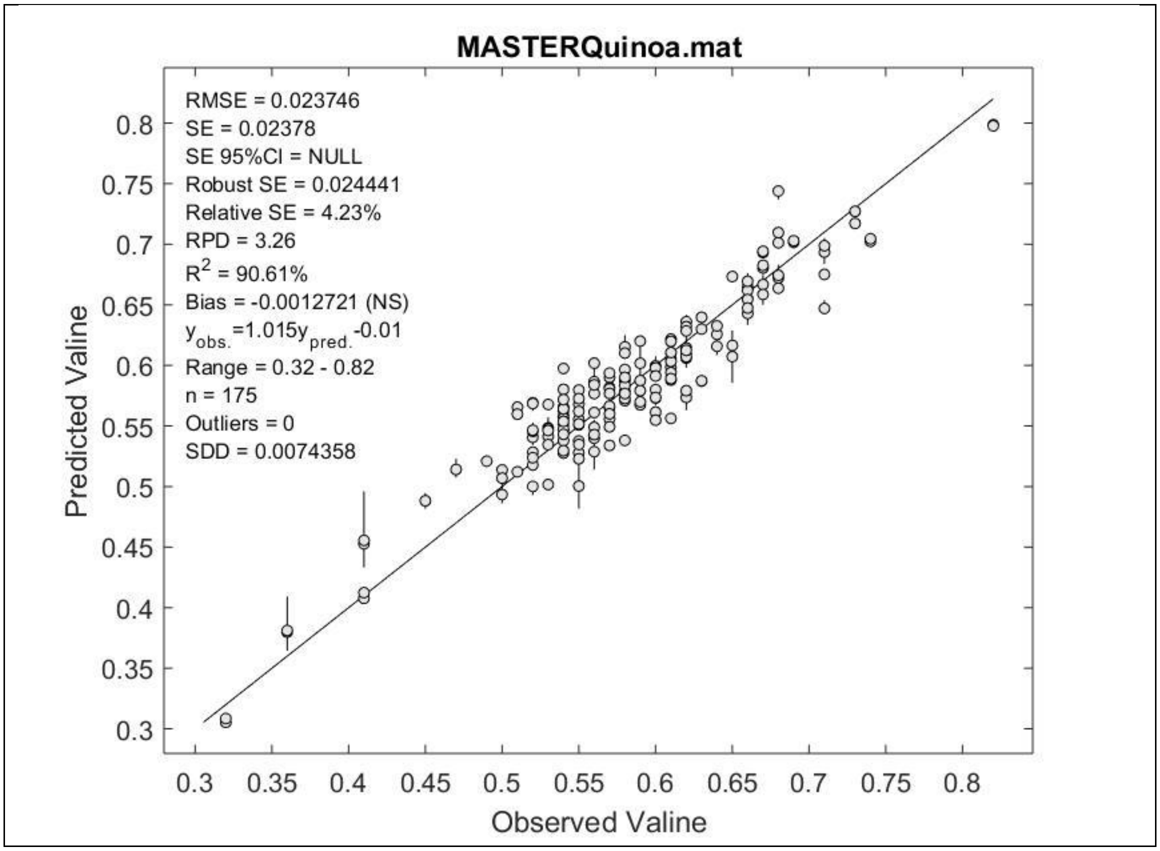

MASTERQuinoa.mat
RMSE = 0.023746
SE = 0.02378
SE 95%CI = NULL
Robust SE = 0.024441
Relative SE = 4.23%
RPD = 3.26
R^2 = 90.61%
Bias = -0.0012721 (NS)
$y_{obs.}$=1.015$y_{pred.}$-0.01
Range = 0.32 - 0.82
n = 175
Outliers = 0
SDD = 0.0074358
Predicted Valine
Observed Valine
0.3
0.35
0.4
0.45
0.5
0.55
0.6
0.65
0.7
0.75
0.8
